# Five new species of *Tetragoniceps* Brady, 1880 (Copepoda, Harpacticoida, Tetragonicipitidae) from Korea

**DOI:** 10.3897/zookeys.1293.206992

**Published:** 2026-09-28

**Authors:** Kyuhee Cho, Jihye Seo, Jong Guk Kim, Jimin Lee

**Affiliations:** 1 Ocean Climate Response & Ecosystem Research Department, Korea Institute of Ocean Science & Technology, Busan 49111, Republic of Korea Ocean Climate Response & Ecosystem Research Department, Korea Institute of Ocean Science & Technology Busan Republic of Korea; 2 Department of Convergence Study on the Ocean Science and Technology, Ocean Science and Technology School, National Korea Maritime & Ocean University, Busan 49112, Republic of Korea Department of Convergence Study on the Ocean Science and Technology, Ocean Science and Technology School, National Korea Maritime & Ocean University Busan Republic of Korea; 3 Division of Zoology, Honam National Institute of Biological Resources, Mokpo 58762, Republic of Korea Division of Zoology, Honam National Institute of Biological Resources Mokpo Republic of Korea

**Keywords:** meiofauna, northwestern Pacific, taxonomy, *Tetragoniceps brevisetosus* sp. nov., *Tetragoniceps concavus* sp. nov., *Tetragoniceps protuberans* sp. nov., *Tetragoniceps spinulifer* sp. nov., *Tetragoniceps unisetus* sp. nov.

## Abstract

Five new species of the genus *Tetragoniceps* Brady, 1880, collected from Korean waters, are described. These new species are readily distinguished from other congeners by a unique armature formula of legs 2–4, notably with one inner seta on the two proximal exopodal segments, and six, five, and seven elements on the distal exopodal segments of legs 2–4, respectively, a combination not previously reported in *Tetragoniceps*. Additional specific diagnostic features of the five new species include the shape and length of the caudal rami, the morphology of leg 5, relative lengths of the inner setae on exopods of legs 2–4, setation of the mandibular exopod, maxillular exopod, and distal endopod segment of leg 1, and sexually dimorphic features of the middle exopodal segment of leg 4 in the male. A comparative table for all known *Tetragoniceps* species, incorporating revised interpretations of several diagnostic characters including the armature formula for the distal exopodal segment of leg 4 in the female, is provided, together with an updated identification key. This study represents the first record of *Tetragoniceps* from the northwestern Pacific, substantially expanding the known geographic distribution of the genus.

## Introduction

The genus *Tetragoniceps* Brady, 1880 was originally established for *Tetragoniceps longiremis* Brady & Robertson, 1876 (Brady and Robertson 1876). Subsequently, Brady (1880) recognized that *T. longiremis* had been misidentified and was in fact *Laophonte thoracica* Boeck, 1865. To accommodate a valid representative of the genus, he described *Tetragoniceps malleolata* Brady, 1880, which was later emended to *T. malleolatus* by Huys (2009). The genus *Tetragoniceps* was originally placed within the Canthocamptidae Brady, 1880, but Lang (1944) subsequently established the family Tetragonicepsidae, with *Tetragoniceps* as the type genus; Wells (1967) later corrected the faulty Latinization of the family name to Tetragonicipitidae Lang, 1944.

To date, 16 *Tetragoniceps* species have been recognized worldwide. Among these, nine species have been reported from the Atlantic Ocean, three from the Pacific Ocean, and four from the Indian Ocean. In the Atlantic Ocean, four species (*T. malleolatus*, *T. brevicauda* Scott T, 1900, *T. scotti* Sars GO, 1911, and *T. bergensis* Por, 1965) occur in the northeastern Atlantic (Brady 1880; Scott 1900; Sars 1911; Por 1965), four (*T. truncata* Nicholls, 1939, *T. longicaudata* Nicholls, 1939, *T. prima* (Coull, 1971), and *T. bookhouti* Coull, 1971) in the northwestern Atlantic (Nicholls 1939; Coull 1971), and one (*T. bermudensis* Mussini, Niimi, Khodami, Kihara, Martinez Arbizu & Blanco-Bercial, 2025) was recently described from anchialine caves in Bermuda (Mussini et al. 2025). All Pacific species (*T. galapagoensis* Mielke, 1989, *T. santacruzensis* Mielke, 1997, and *T. pacificus* Burgess, 1998) were reported from the eastern Pacific (Mielke 1989, 1997; Burgess 1998), whereas the Indian Ocean fauna comprises *T. dubia* Thompson IC & Scott A, 1903, *T. arenicolous* Krishnaswamy, 1957, *T. brownei* Wells, 1967, and *T. unguis* Wells & Rao, 1987 (Thompson and Scott 1903; Krishnaswamy 1957; Wells 1967; Wells and Rao 1987). Despite the broad geographic distribution of the genus, *Tetragoniceps* has not previously been reported from Korean waters.

During a biodiversity study of benthic harpacticoids in Korea, five new *Tetragoniceps* species were discovered in intertidal and sublittoral habitats. This study represents the first record of the genus from Korean waters and substantially expands its known distribution in the northwestern Pacific. We describe these new species and provide a revised comparative table together with an updated identification key for all known species of *Tetragoniceps*.

## Materials and methods

**Figure 1.**
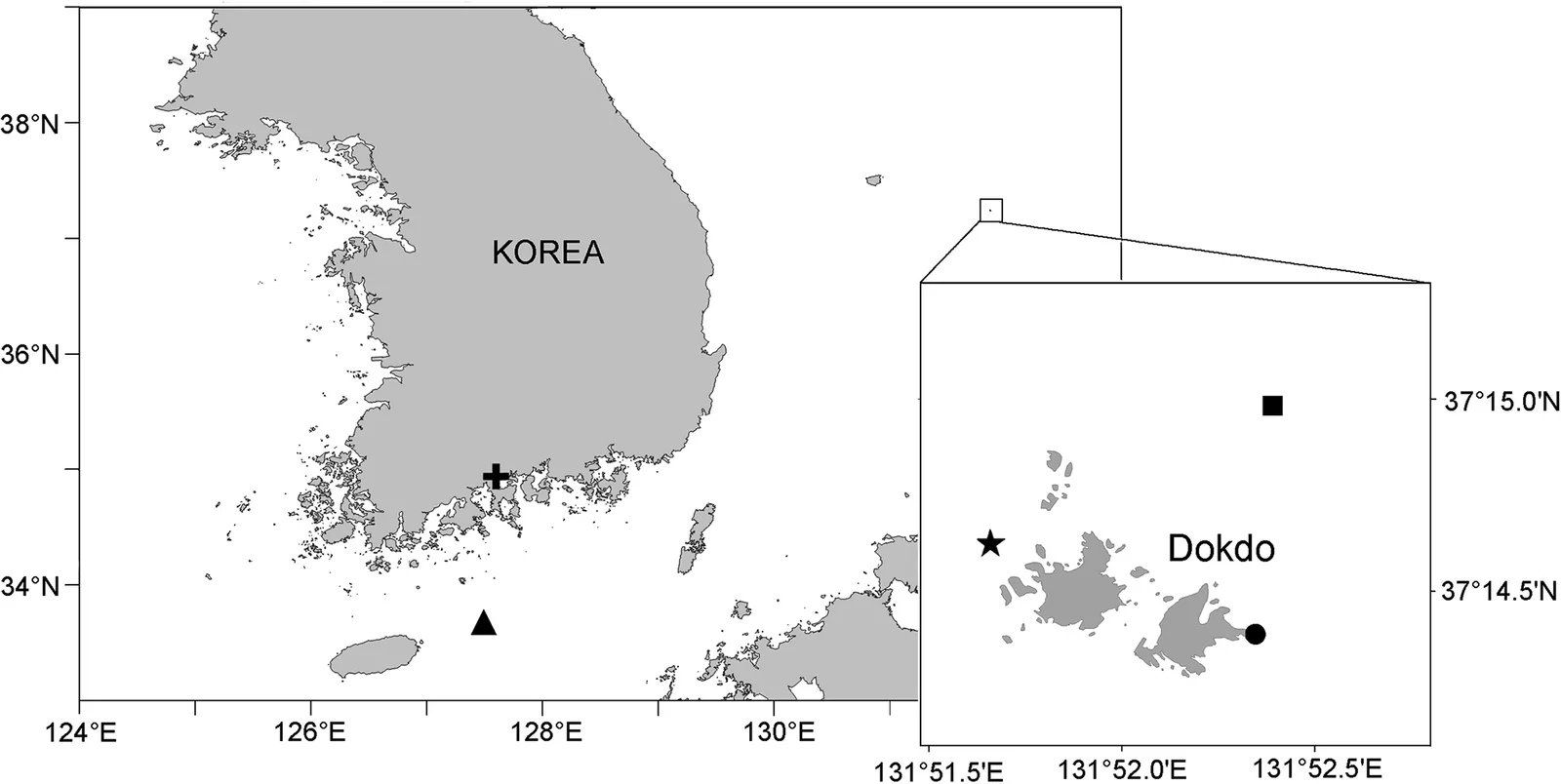
Map of the sampling stations of five new *Tetragoniceps* species. Symbol: star, *T. concavus* sp. nov.; circle, *T. spinulifer* sp. nov.; square, *T. brevisetosus* sp. nov.; triangle, *T. unisetus* sp. nov.; plus, *T. protuberans* sp. nov.

Field samples were collected from five stations around Korea (Fig. 1) using various methods, including SCUBA diving, a Smith-McIntyre grab sampler (0.1 m^2^), and the Karaman-Chappuis method. Each sample was sieved through a hand net (64 μm mesh) and immediately preserved in 95% ethanol or fixed in 10% formalin. Benthic organisms were separated from sediments using Ludox centrifugation (Burgess 2001). In the laboratory, *Tetragoniceps* specimens were sorted using a stereomicroscope (M165 C; Leica, Wetzlar, Germany) and preserved in 80% or 95% ethanol.

Specimens were examined and illustrated using a drawing tube mounted on a BX53 microscope (Olympus, Tokyo, Japan) equipped with differential interference contrast. Dissected appendages were mounted on H-S slides in a lactophenol:glycerol (1:5) mixture, allowing observation from both sides (Shirayama et al. 1993), and the edges of the coverslips were sealed with nail varnish.

The terminology for morphological descriptions followed that of Huys et al. (1996), and the setal armature formulae of thoracic legs were adopted from Lang (1934). The following abbreviations were used: **ae**, aesthetasc; **enp**, endopod; **exp**, exopod; **exp(enp)-1(-2, -3)** to denote the proximal (middle, distal) segment of a ramus; and **P1–P6**, first to sixth legs.

The type materials of the new *Tetragoniceps* species, except for *Tetragoniceps protuberans* sp. nov., were deposited in the Marine Biodiversity Institute of Korea (**MABIK**), Seocheon, Korea; those of *T. protuberans* sp. nov. were deposited in the Honam National Institute of Biological Resources (**HNIBR**), Mokpo, Korea.

## Results

**Order Harpacticoida Sars, 1903**

**Family Tetragonicipitidae Lang, 1944**

**Genus *Tetragoniceps* Brady, 1880**

### Tetragoniceps concavus sp. nov.

Taxon classificationAnimaliaHarpacticoidaTetragonicipitidae

21A46671-1D35-5705-8AF5-963F72EB6CDA

https://zoobank.org/7B484B2F-CBD9-4EB9-BB15-D87CDF871355

Figs 2, 3, 4, 5

#### Type locality.

Dokdo Island, Korea; 37°14'37.23"N, 131°51'39.61"E; depth 15 m.

#### Type material.

***Holotype***. • One ♀ (MABIK CR00261557) preserved in a vial with 80% ethanol, collected from the type locality, 22 June 2016, T.W. Jeong leg. ***Allotype***. • One ♂ (MABIK CR00261558) preserved in a vial with 80% ethanol, collection data as in holotype. ***Paratypes***. • Two ♀♀ (MABIK CR00261559, MABIK CR00261560) and two ♂♂ (MABIK CR00261561, MABIK CR00261562) each dissected on three H-S slides, collection data as in holotype.

#### Description.

**Figure 2.**
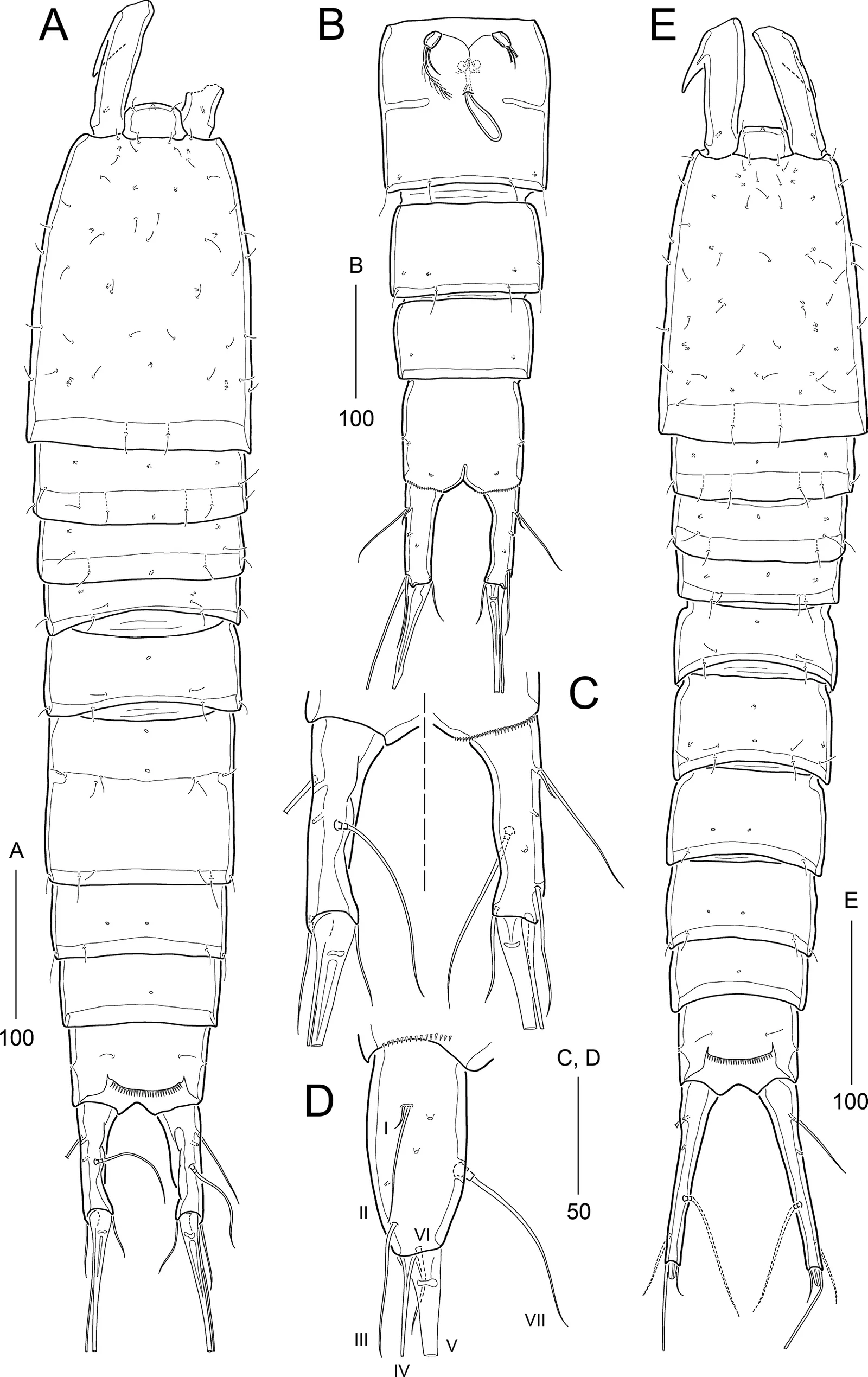
*Tetragoniceps concavus* sp. nov. Holotype (MABIK CR00261557), female. **A**. Habitus, dorsal. Paratype (MABIK CR00261559), female; **B**. Urosome, ventral; **C**. Caudal ramus, dorsal (left) and ventral (right); **D**. Caudal ramus, lateral. Allotype (MABIK CR00261558), male; **E**. Habitus, dorsal. Scale bars are in μm.

**Female**. Body length range 795–959 μm (*n* = 3, mean = 876 μm, holotype = 874 μm) measured from anterior tip of rostrum to posterior end of caudal rami (in lateral view). Habitus (Fig. 2A) subcylindrical, without clear demarcation between prosome and urosome. Prosome (Fig. 2A) slightly shorter than urosome, comprising cephalothorax (cephalosome completely fused with first pedigerous somite) and three free pedigerous somites; dorsal surface ornamented with integumental pores and sensilla as figured (Fig. 2A). Cephalothorax (Fig. 2A) ~ 1.4× as long as wide, greatest width 157 μm measured at posterior end of cephalothorax.

Urosome (Fig. 2A, B) comprising P5-bearing somite, genital double-somite, and three free abdominal somites. Genital somite and first abdominal somite fused, forming genital double-somite; original segmentation indicated dorsally by a faint line on dorsal surface and internal chitinous ribs on ventrolateral regions; ornamentation composed of ventral and dorsal pores and sensilla as figured (Fig. 2A, B). Genital field (Figs 2B, 4F) located medially in anterior half of genital double-somite. Genital apertures fused to median V-shaped slit. Copulatory pore located at approximately midpoint of double-somite length (Fig. 2B). First free abdominal somite (Fig. 2A, B) slightly longer than second free abdominal somite; with dorsal and ventral sensilla and pores. Second free abdominal somite (Fig. 2A, B) with pores only. Anal somite (Fig. 2A, B) longer than penultimate somite, with one pair of dorsal sensilla, one pair of ventral pores, and two pairs of ventrolateral pores; posteroventral margin with minute spinules; anal operculum semicircular, posterior margin with fine spinules.

Caudal ramus (Fig. 2A–D) cylindrical, ~ 2.6× as long as maximum width in dorsal aspect and 2.1× as long as wide in lateral view; dorsomedial surface slightly concave and constricted near insertion of seta VII, but cylindrical in ventral view; with a weak ventroposterior protuberance and three pores, one each on dorsal, ventral, and lateral surfaces; with seven setae: setae I and II positioned on anterior 1/3 of lateral margin; seta I diminutive; seta II slender and naked, 5× longer than seta I; seta III arising from anterior 4/5 of lateral margin, slightly shorter than seta II; setae IV and V fused basally, the former slender; seta V longest; seta VI ~ 1/2 length of seta II; seta VII tri-articulated, inserted dorsally at mid-length.

Rostrum (Fig. 2A) small, subrectangular with rounded corners, completely defined basally, with one pair of subapical sensilla and one apical median pore.

**Figure 3.**
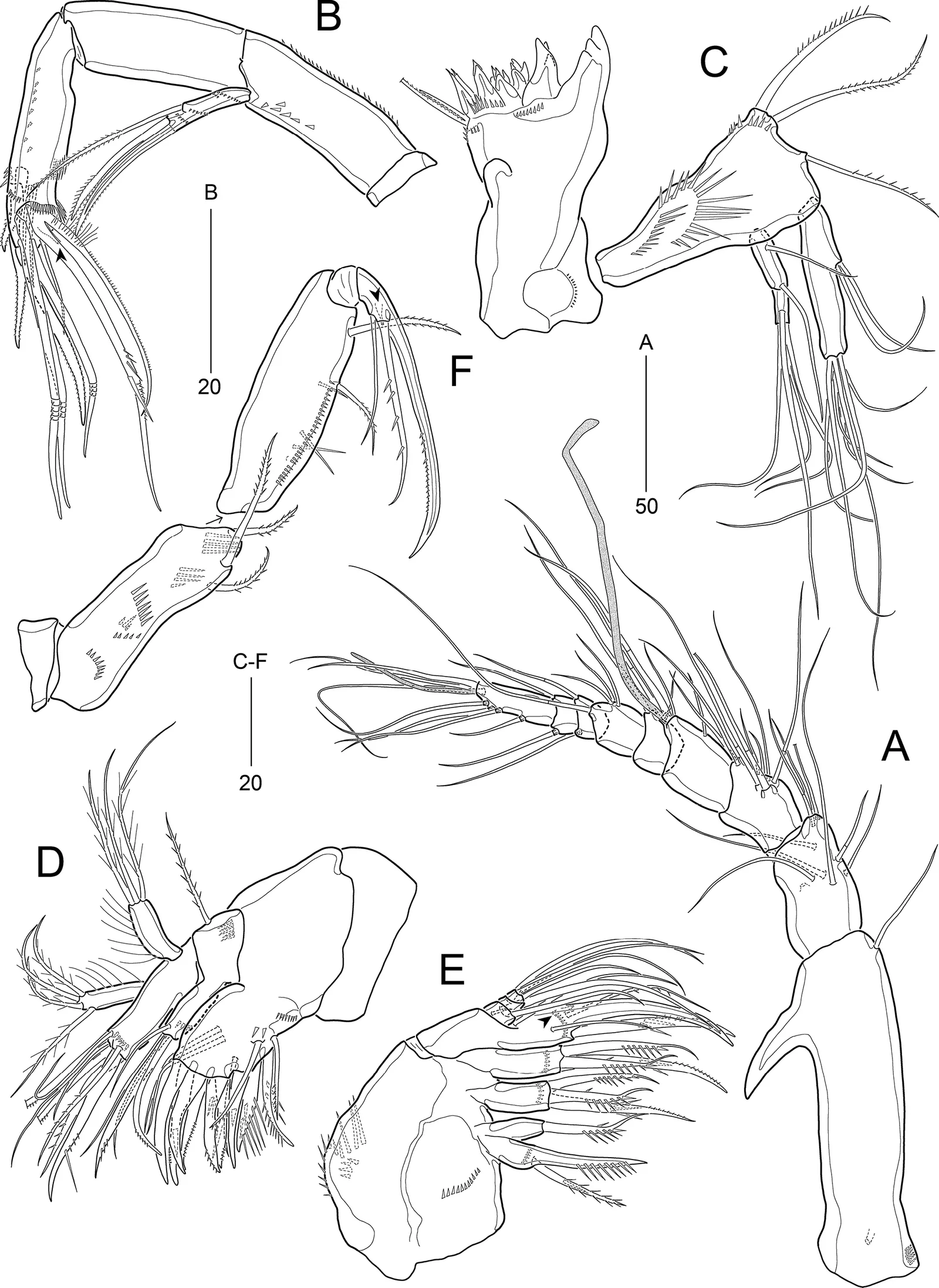
*Tetragoniceps concavus* sp. nov. Paratype (MABIK CR00261559), female. **A**. Antennule; **B**. Antenna; **C**. Mandible; **D**. Maxillule; **E**. Maxilla; **F**. Maxilliped. Scale bars are in μm.

Antennule (Fig. 3A) nine-segmented; first segment elongated, ~ 3× as long as second segment; with strong acute process on outer distal corner, a proximal pore, and several minute inner spinules, and one naked seta; second segment with nine naked setae and a pore; fourth segment with aesthetasc fused basally to one long seta on inner distal corner; seventh and eighth segments small, each with one bi-articulate and one naked seta; ninth segment elongate, with four bi-articulate setae, one naked seta, and apical acrothek consisting of two setae and slender aesthetasc. Armature formula as follows: 1-[1], 2-[9], 3-[8], 4-[3 + (1 + ae)], 5-[2], 6-[4], 7-[2], 8-[2], 9-[5 + acrothek].

Antenna (Fig. 3B). Coxa small, unornamented. Basis elongated, ornamented with rows of spinules along inner and outer margins. Exopod arising distally on basis, one-segmented, ~ 4× as long as wide; ornamented with several rows of minute spinules, and with three pinnate setae (outer distal seta incompletely fused to the exopod), outer distal seta slightly longer than inner distal seta. Endopod two-segmented; first segment as long as basis, unarmed; second segment as long as preceding segment, ornamented with inner and distal spinules, and two hyaline frills (one subdistal and one distal); laterally with two naked setae and two bipinnate spines; distally with one tube pore (indicated by arrowhead in Fig. 3B) and seven elements (one bipinnate spine, one pinnate, one naked, and four geniculate pinnate setae), of which two outermost setae (one geniculate pinnate and one pinnate) fused at base.

Mandible (Fig. 3C). Coxa with a rounded protuberance medially; gnathobase with two tri-cuspidate and several bi- and multi-cuspidate teeth, and one unipinnate seta fused to one small spine; subdistally ornamented with three rows of spinules. Palp biramous; basis elongated, gradually widening towards medial margin, with two rows of long spinules on surface, several spinules on disto-medial corner, and three subequal pinnate setae along inner margin. Exopod two-segmented, extending beyond half the length of endopod; first segment longer than second, with one proximal and one distal seta; distal segment with one subapical and three apical setae. Endopod one-segmented, elongated, with two lateral and seven distal setae, including two pairs of basally fused setae.

Maxillule (Fig. 3D). Praecoxal arthrite well-developed, with two juxtaposed and naked anterior setae, eight distal spines, one naked subdistal seta, one lateral pinnate seta, and two pinnate posterior setae. Coxa with one epipodal seta and one row of spinules anteriorly; cylindrical endite with subdistal spinular row and five distal elements (one spine and four setae). Basis elongate, ornamented with subdistal spinular row, and with one spine and seven setae medially. Exopod elongate, with inner setules and three distal bipinnate setae. Endopod as long as exopod, with outer setules and four pinnate setae (three distal and one subdistal).

Maxilla (Fig. 3E). Syncoxa large, ornamented with several spinules along outer margin and a row of spinules on anterior surface; with four endites: proximal praecoxal endite with a subdistal spinular row, one bipinnate seta and one pinnate spine (fused to endite); distal praecoxal endite with one pinnate seta; proximal and distal coxal endites each with a row of spinules on posterior surface subdistally and three pinnate setae distally. Allobasis drawn out into a curved claw accompanied by one tube pore (indicated by arrowhead in Fig. 3E), with three subdistal setae (one short naked and two long pinnate), and one proximal naked seta; with a subdistal spinular row on posterior surface. Endopod small, three-segmented; first segment with three setae, one of these very short; second segment with one seta; third segment with three setae.

Maxilliped (Fig. 3F) subchelate, arising from small pedestal. Syncoxa with several spinular rows and three pinnate setae on inner distal corner. Basis longer than syncoxa, with several long spinules and a row of minute spinules, and two pinnate setae along inner margin. Endopod drawn out into strong, slightly curved claw bearing minute spinules in distal half; one anterior tube pore (indicated by arrowhead in Fig. 3F); accessory armature consisting of one pinnate seta and one naked seta.

**Figure 4.**
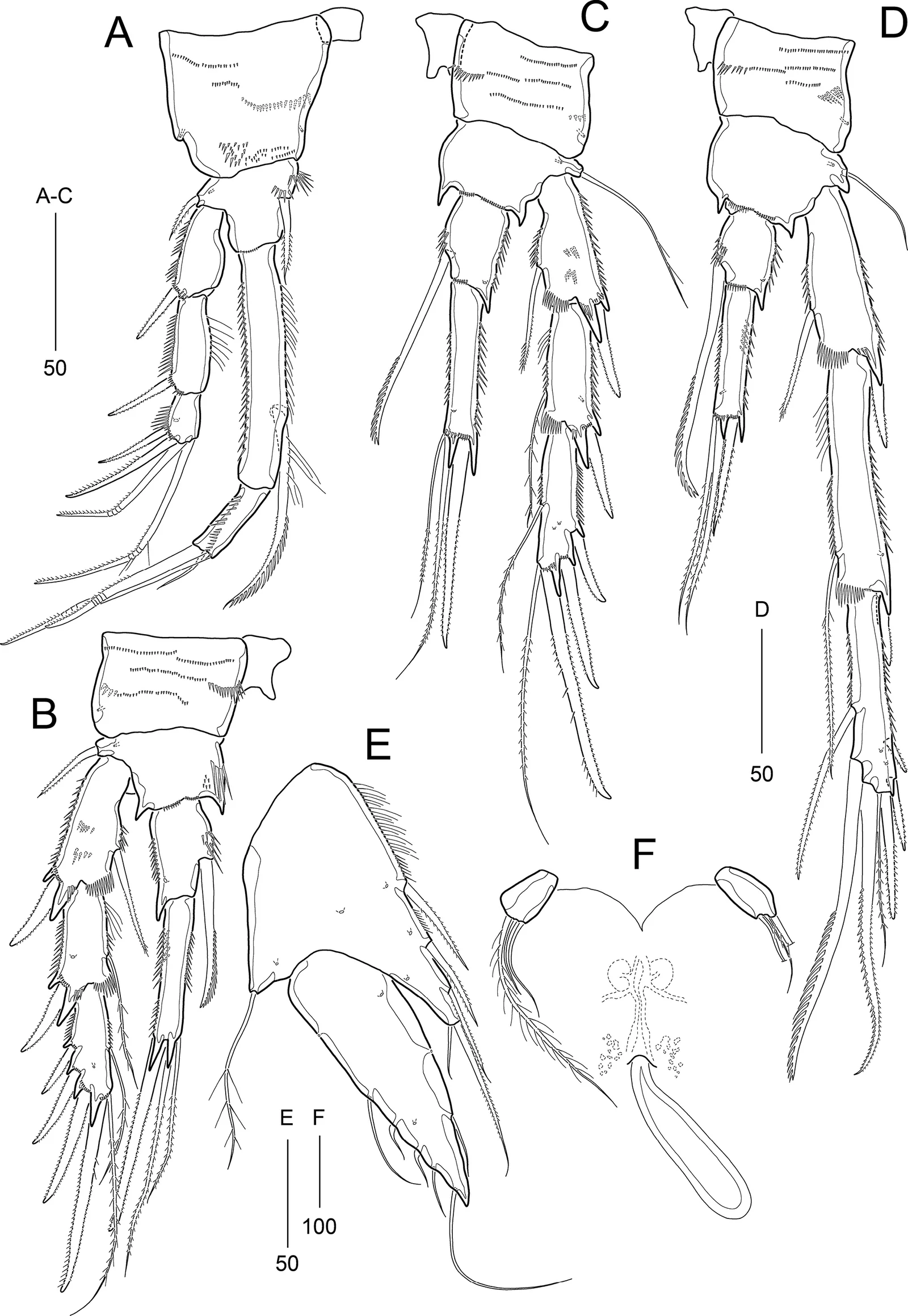
*Tetragoniceps concavus* sp. nov. Paratype (MABIK CR00261559), female. **A**. P1, anterior; **B**. P2, anterior; **C**. P3, anterior; **D**. P4, anterior; **E**. P5, anterior; **F**. Genital field. Scale bars are in μm.

P1 (Fig. 4A). Intercoxal sclerite small, trapezoidal. Praecoxa not illustrated. Coxa large, with rows of anterior and posterior spinules as figured, and one anterior pore on inner margin and another on outer margin. Basis distinctly smaller than coxa, with two rows of spinules on inner margin and one anterior pore near outer margin; with one outer and one inner bipinnate spine. Exopod three-segmented, each segment with outer marginal spinules; exp-1 and exp-2 with outer bipinnate spines, respectively; exp-2 elongate, with inner setules; exp-3 smallest, with one posterior pore and four elements (two outer pinnate spines and two distal geniculate pinnate setae). Endopod distinctly longer than exopod, two-segmented; enp-1 elongated, as long as exp-1 to exp-3 combined, 2.6× as long as enp-2, with inner setules, outer spinules, and one inner seta with proximal half bearing long spinules and distal half pectinate on one margin and pinnate on the other, and extending far beyond tip of enp-2; enp-2 ~ 3.6× as long as wide, with a row of spinules and three setae (two geniculate pinnate and one short naked).

P2 (Fig. 4B). Intercoxal sclerite nearly rectangular. Praecoxa not illustrated. Coxa large, with several rows of anterior and posterior spinules as figured and one anterior pore near outer margin. Basis with inner marginal spinules, one anterior pore, and one outer pinnate spine; distal margin with two small acute processes (inner and median) and minute marginal spinules between two processes. Exopod three-segmented, each segment ornamented with inner and outer marginal spinules; exp-1 and exp-2 each with inner distal hyaline frill and one outer bipinnate spine; exp-1 with posterior spinules and one inner bipinnate seta; exp-2 with inner setules proximally, one anterior pore, and one inner pinnate seta; exp-3 with one anterior pore, one inner pinnate seta, one apical pinnate spine, one apical pinnate seta, and three outer pinnate spines; inner setae on exp-2 and exp-3 subequal in length, slightly longer than terminal segment length. Endopod two-segmented, reaching mid-length of exp-3; each segment ornamented with inner and outer spinules; enp-1 with one anterior pore and one inner unipectinate seta; enp-2 longer and slenderer than preceding segment, with one outer pinnate spine and two apical pinnate setae; outer spine elongate, longer than supporting segment (enp-2); distal margin with two distinct processes.

P3 (Fig. 4C). Intercoxal sclerite, coxa, and basis as in P2, but basis lacking inner spinules and bearing one elongate and plumose outer seta. Exopod and endopod as in P2, except exp-3 with two anterior pores and only two outer pinnate spines, and enp-2 with one anterior pore; inner seta on enp-1 slightly exceeding distal end of ramus.

P4 (Fig. 4D). Intercoxal sclerite, coxa, and basis as in P3, but outer seta on basis naked and shorter than that on P3. Exopod distinctly elongate, ~ 2.5× as long as endopod; ornamentation of segments as in P2 and P3, but exp-1 without posterior spinules; exp-2 elongate, with one inner bipinnate seta; exp-3 with two anterior pores as in P3 and seven pinnate elements (three inner, two apical, and two outer), of the three inner elements, the two distal ones robust and long; proximal one unipectinate and distal one bipinnate. Endopod as in P2 and P3, but inner seta on enp-1 distinctly exceeding distal end of ramus and outer spine on enp-2 distinctly shorter than enp-2 length, as long as half-length of adjacent distal setae.

Armature formula of P1–P4 as follows:

**Table:** 

	Exopod	Endopod
P1	0.0.022	1.120
P2	1.1.123	1.021
P3	1.1.122	1.021
P4	1.1.322[1.1.222 in ♂]	1.021

P5 (Fig. 4E). Baseoendopod and exopod distinct. Baseoendopod consisting of broadened proximal half and triangular distal half; inner margin ornamented with setules in proximal three-quarters and anterior surface with five pores; outer margin extended outwards, with plumose (basal) seta; endopodal lobe with three inner bipinnate setae and two apical setae, of which outer one shortest, naked, and inner one longest, bipinnate, reaching tip of exopod. Exopod elongate, gradually tapering to pointed tip in distal half, ~ 3.1× as long as wide; with one inner and five outer setae, of which second and third outer proximal setae short and basally swollen, and fifth outer seta longest; with one inner and one outer marginal pore, and two anterior pores.

P6 (Fig. 4F) represented by a small segment bearing one plumose and two naked setae; outermost (plumose) seta ~ 2× as long as innermost seta, and middle seta shortest.

**Male**. Body length range 709–835 μm (*n* = 3, mean = 778 μm; measurement as in females). Habitus (Fig. 2E) as in females, except for urosomal segmentation. Sexual dimorphism observed in antennule, urosome, caudal rami, and P2–P6.

**Figure 5.**
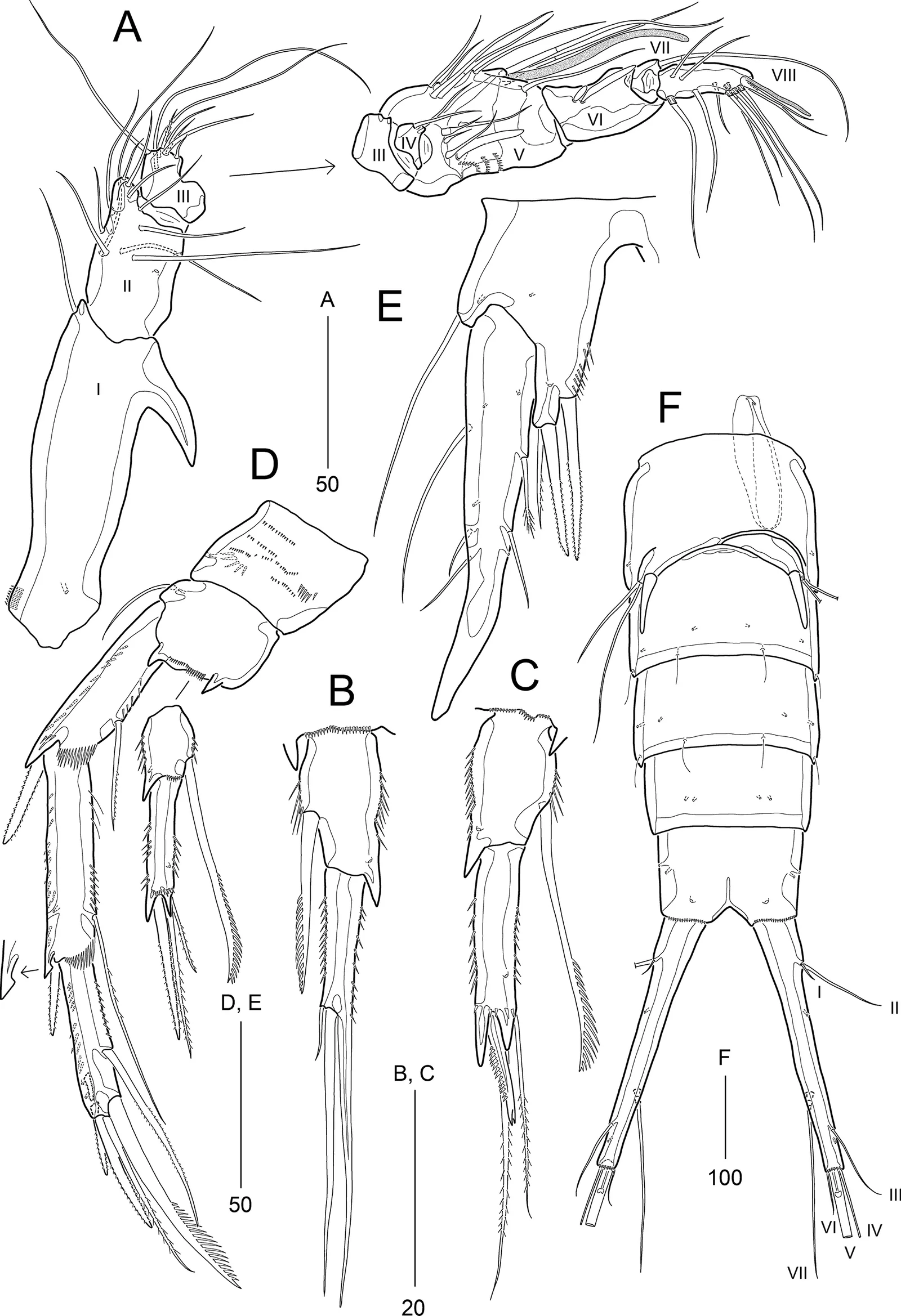
*Tetragoniceps concavus* sp. nov. Paratype (MABIK CR00261561), male. **A**. Antennule; **B**. P2 endopod, anterior; **C**. P3 endopod, anterior; **D**. P4, anterior; **E**. P5, anterior; **F**. Urosome, ventral. Scale bars are in μm.

Urosome (Figs 2E, 5F) six-segmented; genital somite and first abdominal somite completely separated, with pores and sensilla as figured.

Caudal rami (Figs 2E, 5F) cylindrical, tapering distally, ~ 6.5× as long as maximum width; with seven setae; setae I and II positioned on anterior 1/5 of lateral margin; seta III arising from posterior 1/5 of lateral margin; seta VII inserted dorsally at posterior 1/3 of ramus.

Antennule (Fig. 5A) eight-segmented, subchirocer, with geniculation between fifth and sixth segments; first segment as in females; fourth segment smallest; fifth segment swollen, with several rows of spinules and faint suture lines; sixth segment with two unequal elements. Armature formula: 1-[1], 2-[11], 3-[8], 4-[2], 5-[11 + 1 element + (1 + ae)], 6-[2 elements], 7-[1], 8-[9 + acrothek].

P2 exopod as in females, except inner setae on exp-2 and exp-3 longer than those in females. Enp-2 (Fig. 5B) shorter than in females, as long as preceding segment, with one apophysis (basally fused to segment) and two naked setae (inner short and outer long); distal margin without protrusions.

P3 exopod as in females, except inner setae on exp-2 and exp-3 longer than those in the females. Enp-2 (Fig. 5C) ~ 1.3× as long as preceding segment, with one short outer spine bearing unilateral spinules and with bifid tip and two long distal bipinnate setae; outer spine half as long as inner apical seta.

P4 (Fig. 5D) as in females, but exp-2 with modified outer distal corner with concave inner margin and blunt tip (shown separately in Fig. 5D); exp-3 with six elements (two inner, two apical, and two outer). Endopod shorter than in females; enp-2 with inner seta distinctly shorter than that in females and outer spine slightly shorter than outer apical seta.

P5 (Fig. 5E). Baseoendopods fused medially, each with three anterior pores and one outer basal seta; endopodal lobe reaching proximal 1/4 of exopod, with inner marginal spinules, one subdistal bipinnate spine, one distal bipinnate spine, and one distal pinnate seta, of which outermost seta shorter than other spines. Exopod one-segmented, ~ 6× as long as maximum width; with distal end produced into a long extension; with one inner marginal and three anterior pores, and three outer and two inner unequal setae (outer proximal seta longest, ~ 1/2 length of exopod, and inner proximal seta bipinnate distally).

P6 (Fig. 5F) asymmetrical, left plate functional, the other fused to genital somite; outer distal corner bearing one stout spine (innermost) and two slender setae (outer one ~ 3/5 length of inner one).

#### Etymology.

The specific epithet *concavus* is derived from the Latin adjective *concavus* (concave) and refers to the slightly concave dorsomedial surface of the female caudal ramus. It is in the nominative singular. Gender masculine.

#### Remarks.

*Tetragoniceps concavus* sp. nov. and four other new species described herein were assigned to *Tetragoniceps* based on the presence of an elongate first antennulary segment bearing a strong, acute outer process and a non-foliaceous female P5.

The diagnostic characters traditionally used to distinguish *Tetragoniceps* species (Wells 2007; Mussini et al. 2025) include the segmentation of the female antennule, the number of setae on the antennary exopod, the segmentation of the mandibular exopod, the shape of the caudal rami, the morphology of the female P5, and the armature formulae of swimming legs. *Tetragoniceps concavus* sp. nov. is readily distinguished from all known congeners by the unique chaetotaxy of its swimming legs, having a single inner seta on exp-1 and exp-2 of P2–P4 and six, five, and seven elements on exp-3 of P2–P4, respectively. This combination of armature patterns has not been observed in previously described congeners.

The species is further distinguished by the shape of the female caudal ramus, with the inner margin irregularly dentate, concave, and constricted at mid-length. In dorsal view, the caudal ramus is cylindrical and 2.6× as long as wide, whereas in lateral view it appears comparatively broader, being 2.1× as long as wide. The male caudal ramus exhibits sexual dimorphism, being considerably more elongate than those of the female and approximately 6.5× as long as wide.

### Tetragoniceps spinulifer sp. nov.

Taxon classificationAnimaliaHarpacticoidaTetragonicipitidae

8A4A8928-30E5-5D3F-B22C-FE48A0DC21AA

https://zoobank.org/7A17A425-79F3-4D0C-A30F-0B81F95BB00D

Figs 6, 7, 8, 9

#### Type locality.

Dokdo Island, Korea; 37°14'23.28"N, 131°52'20.64"E; depth 25 m.

#### Type material.

***Holotype***. • One ♀ (MABIK CR00261563) preserved in a vial with 80% ethanol; collected from the type locality, 28 May 2015, H.S. Rho leg. ***Allotype***. • One ♂ (MABIK CR00261564) preserved in a vial with 80% ethanol, collection data as in holotype. ***Paratypes***. • Three ♀♀ (MABIK CR00261565–CR00261567) and three ♂♂ (MABIK CR00261568–CR00261570) each dissected on three H-S slides, collection data as in holotype.

#### Description.

**Figure 6.**
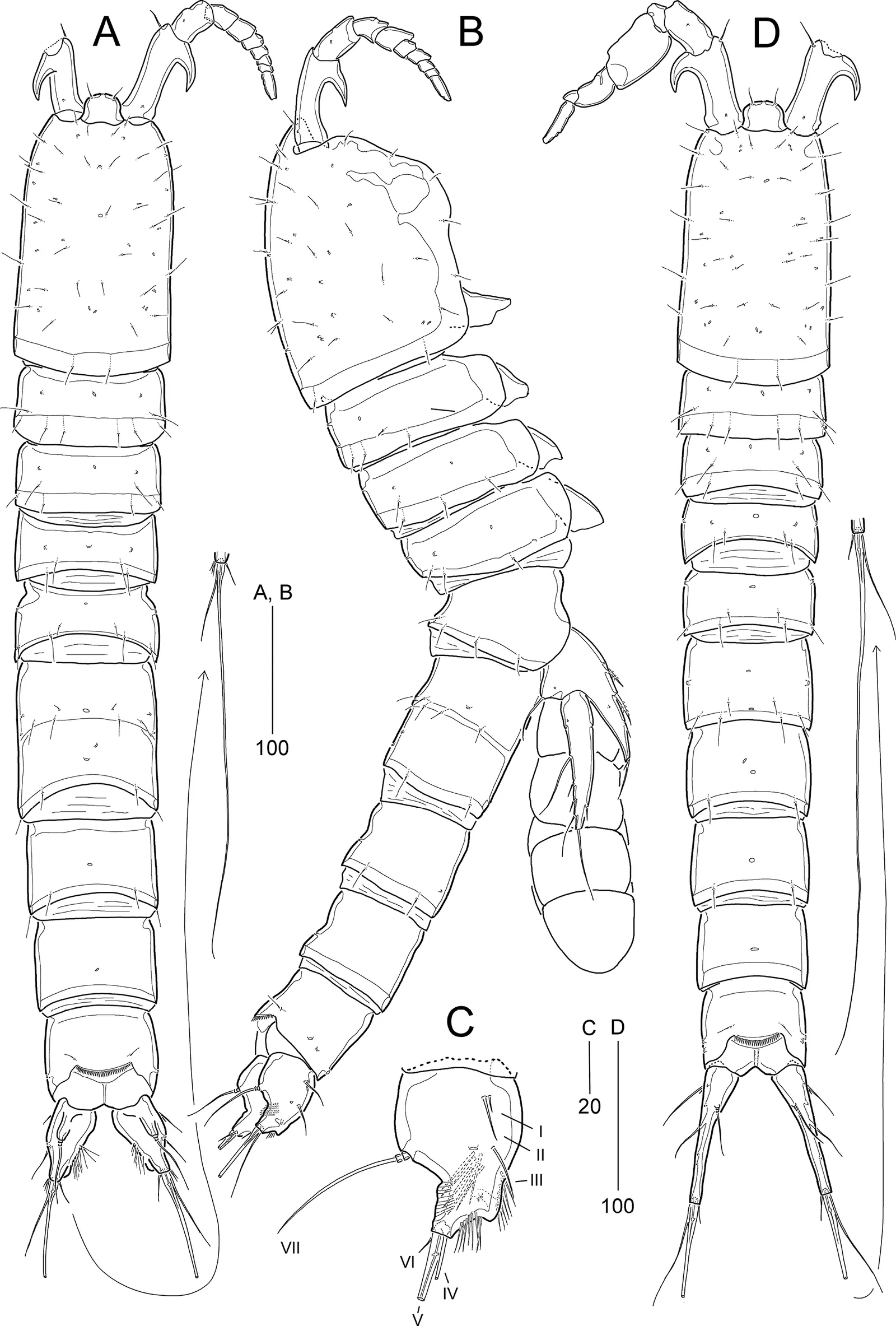
*Tetragoniceps spinulifer* sp. nov. Holotype (MABIK CR00261563), female. **A**. Habitus, dorsal; **B**. Habitus, lateral; **C**. Caudal ramus, lateral. Allotype (MABIK CR00261564), male; **D**. Habitus, dorsal. Scale bars are in μm.

**Female**. Body length range 625–846 μm (*n* = 4, mean = 744 μm, holotype = 846 μm). Habitus (Fig. 6A, B) subcylindrical, without clear demarcation between prosome and urosome, slenderer than body shape of *T. concavus* sp. nov. Ornamentation composed of numerous integumental pores, small pits (not figured) and sensilla on prosome as figured (Fig. 6A, B). Cephalothorax (Fig. 6A) ~ 1.6× as long as wide, greatest width 122 μm measured at posterior end of cephalothorax.

Genital double-somite (Figs 6A, 6B, 9A) completely fused, original segmentation marked by faint dorsal line and internal chitinous ribs dorsolaterally, laterally, and ventrally. Paired genital apparatus located at anterior 1/6 of genital double-somite; genital pores closed off by reduced P6 (separated from each other), each bearing one long outer plumose seta (longest), one middle naked seta (shortest), and one long inner naked seta; outer seta ~ 1.5× as long as inner seta, and middle seta ~ 1/6 length of inner seta (Fig. 9A). First and second free abdominal somites (Figs 6A, 6B, 9A) subequal in length. Anal somite (Figs 6A, 6B, 9A) slightly smaller than penultimate somite, with one pair of dorsal sensilla, one pair of ventral pores, and two pairs of ventrolateral pores; anal operculum semicircular, ventroposterior margin with minute spinules; anal opening wide.

Caudal rami (Figs 6A–C, 9A) divergent, ~ 1.6× as long as wide in dorsal view, with posterior 2/5 distinctly narrower than anterior 3/5 in lateral view (Fig. 6B, C); ornamented with four pores (Figs 6C, 9A), one row of inner long spinules, and two rows of ventroposterior spinules; with seven setae: setae I and II positioned on anterior 1/5 of lateral margin; seta I diminutive; seta II slender and naked, 2.5× length of seta I; seta III arising from 1/2 on outer margin, as long as seta II; setae IV and V fused basally; seta IV slender, ~ 1.5× as long as caudal ramus; seta V longest; seta VI ~ 2/3 length of seta II; dorsal seta VII tri-articulated basally, inserted in mid-length of ramus.

Rostrum (Fig. 6A) small, separated basally, with one pair of subapical sensilla and one apical median pore.

**Figure 7.**
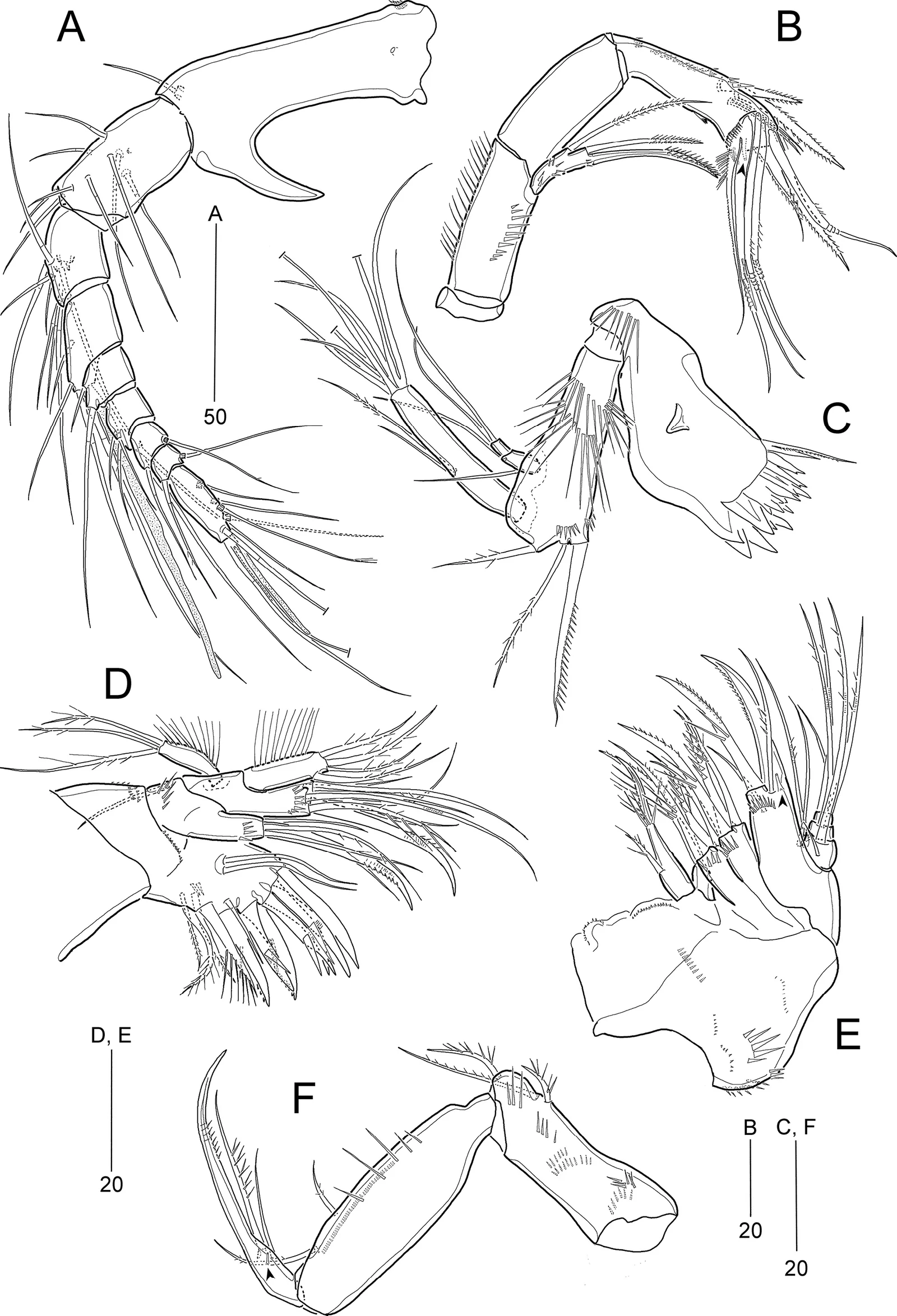
*Tetragoniceps spinulifer* sp. nov. Paratype (MABIK CR00261565), female. **A**. Antennule; **B**. Antenna; **C**. Mandible; **D**. Maxillule; **E**. Maxilla; **F**. Maxilliped. Scale bars are in μm.

Antennule (Fig. 7A) nine-segmented and armature formula as in *T. concavus* sp. nov., but morphological differences detected in the following: first segment ~ 2× as long as second segment; outer protrusion more developed than that of preceding species.

Antenna (Fig. 7B) composed of small coxa, basis, two-segmented endopod, and one-segmented exopod, and armature formula as in *T. concavus* sp. nov., but morphological difference detected in the following: exopod ~ 3.5× as long as wide, smaller than in *T. concavus* sp. nov., with three pinnate setae, outer distal seta longer than inner distal seta.

Mandible (Fig. 7C), maxillule (Fig. 7D), maxilla (Fig. 7E), and maxilliped (Fig. 7F) similar to *T. concavus* sp. nov., but morphological difference detected in the following: mandibular coxa with one row of outer spinules proximally; mandibular palp with three armature elements (most dorsal one uniserrate, spine-like, and longest); mandibular exopod small, 1/3 length of endopod, first segment with one lateral and one subdistal seta, and second segment with two apical setae; maxillular exopod slenderer, but endopod larger than that of *T. concavus* sp. nov.; maxilla with additional ornamentation of minute spinules on syncoxa, pinnate seta on proximal praecoxal endite much smaller than apical spine (fused to endite basally), and two setae on enp-1, one seta on enp-2, and two setae on enp-3, all unipinnate; syncoxa of maxilliped with one unipinnate (middle and longest) and two bispinulose setae, tip of reflexed endopod reaching proximal 1/6 of basis.

Morphological features of P1–P4 (segmentation and ornamentation) as in *T. concavus* sp. nov.

**Figure 8.**
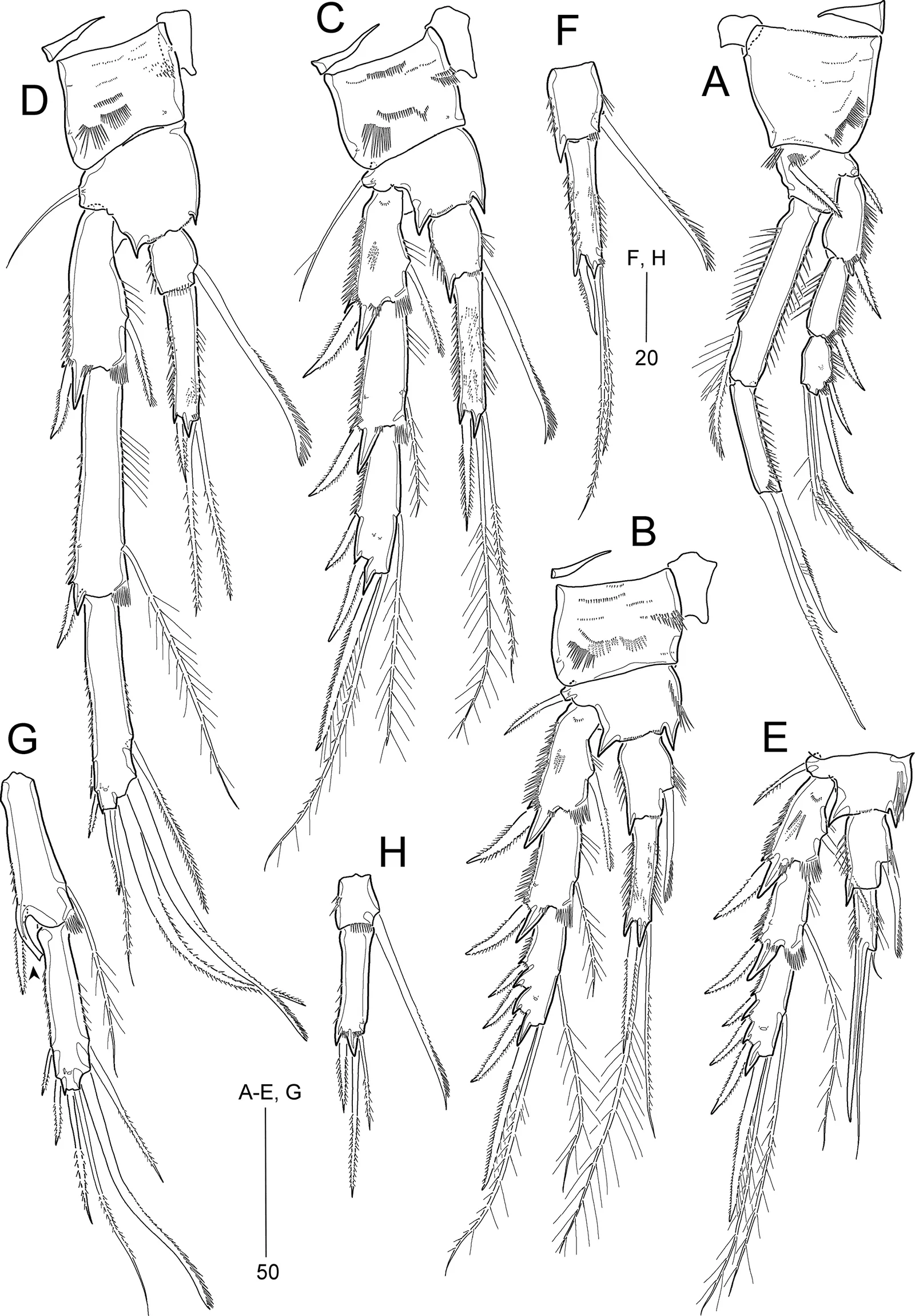
*Tetragoniceps spinulifer* sp. nov. Paratype (MABIK CR00261565), female. **A**. P1, anterior; **B**. P2, anterior; **C**. P3, anterior; **D**. P4, anterior. Paratype (MABIK CR00261568), male; **E**. P2 (omitted coxa), anterior; **F**. P3 endopod, anterior; **G**. P4 exopod, second and third segments, anterior; **H**. P4 endopod. Scale bars are in μm.

P1 (Fig. 8A). Praecoxa triangular, without ornamentation. Coxa with two rows of spinules, several rows of denticles, and one pore on anterior surface. Enp-1 shorter than exopod, 1.6× as long as enp-2, inner seta of enp-1 reaching mid-length of enp-2; enp-2 slender, 6× as long as wide, with only two geniculate pinnate setae (delicate element lacking).

P2 (Fig. 8B). Praecoxa small, transversely elongated. Coxa with two groups of spinules, several rows of denticles and one pore on anterior surface, and two rows of spinules on posterior surface. Two pointed projections on basis distinctly developed. Exp-1 with two rows of spinules (proximal one rounded) on posterior margin; inner seta on exp-3 elongate, 2× as long as that of exp-2. Endopod slightly exceeding tip of exp-2; enp-1 lacking anterior pore; enp-2 as long as exp-2, with several rows of posterior spinules; outer spine on enp-2 ~ 3/5 length of inner distal seta, and outer distal seta elongate (as long as exopod) and plumose.

P3 (Fig. 8C). Praecoxa small, transversely elongated. Coxa with two anterior pores, several rows of spinules and two rows of denticles on anterior surface. Inner seta on exp-3 ~ 2× as long as that of exp-2. Endopod not reaching distal end of exp-2; enp-2 with several rows of posterior spinules, outer spine distinctly shorter than supporting segment, 1/3 length of plumose outer distal seta.

P4 (Fig. 8D). Intercoxal sclerite narrower than in P2 and P3, rectangular. Praecoxa small, transversely elongated. Coxa with three anterior pores, one patch of spinules, three rows of spinules, and several rows of denticles on anterior surface. Two acute processes on basis smaller than those of P2 and P3. Exopod distinctly elongated; exp-2 with one inner plumose seta, ~ 1.2× as long as exp-2, inner margin ornamented with only one row of setules; exp-3 with one small and one large pore on anterior surface, and seven pinnate elements (three inner, two apical, and two outer); two inner distal elements robust and long; outer and apical elements comparatively slenderer than corresponding elements in P2 and P3. Enp-1 small, without inner ornamentation and acute process at outer distal corner; enp-2 with one anterior pore and small outer spine, ~ 1/3 length of apical pinnate setae.

Armature formula of swimming legs as in *T. concavus* sp. nov., except for P1 endopod with 1.020.

**Table:** 

	Exopod	Endopod
P1	0.0.022	1.020
P2	1.1.123	1.021
P3	1.1.122	1.021
P4	1.1.322[1.1.222 in ♂]	1.021

**Figure 9.**
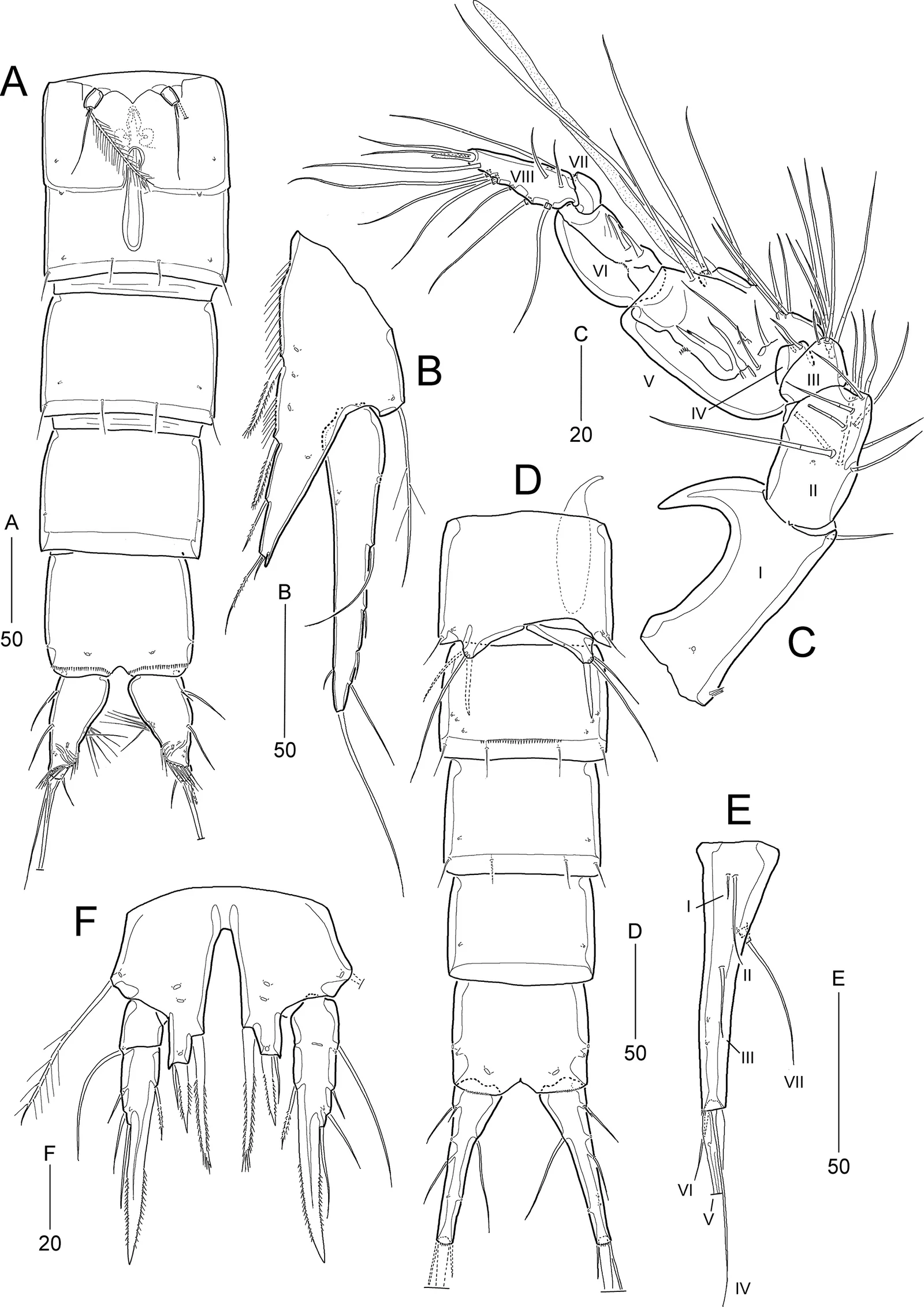
*Tetragoniceps spinulifer* sp. nov. Paratype (MABIK CR00261565), female. **A**. Urosome, ventral; **B**. P5, anterior. Paratype (MABIK CR00261568), male; **C**. Antennule; **D**. Urosome, ventral; **E**. Caudal ramus, lateral; **F**. P5, anterior. Scale bars are in μm.

P5 (Fig. 9B). Baseoendopod and exopod distinct, both more elongate and slenderer than in *T. concavus* sp. nov. Baseoendopod gradually broadening distally, with one outer plumose seta, and ornamented with setules in proximal 2/3 of inner margin and five pores on anterior surface; endopodal lobe triangular in shape, reaching mid-length of exopod; outer distal corner of endopodal lobe with one minute process; with three inner bipinnate setae (distal one slender), one long apical pinnate seta, and one short apical naked seta. Exopod elongate, slender, ~ 5× as long as wide; with one minute inner seta, one long apical seta (2/3 length of exopod), and four outer slender setae, of which second and third proximal setae short; ornamentation with two inner and two outer proximal pores.

**Male**. Body length range 712–730 μm (*n* = 4, mean = 724 μm). Habitus (Fig. 6D) as in females, except for antennule, urosome, caudal rami, and P2–P6.

Urosome (Figs 6D, 9D) with pores and sensilla as ﬁgured; genital somite and first abdominal somite completely separate. First abdominal somite with minute spinular row on posteroventral surface (Fig. 9D).

Caudal rami (Figs 6D, 9D, 9E) divergent, cylindrical, tapering distally, ~ 3.5× as long as wide, with seven setae: seta III arising from mid-length of lateral margin; seta VII (Fig. 9E) inserted dorsally at anterior 1/3 length of ramus.

Antennule (Fig. 9C) eight-segmented, subchirocer, with geniculation between fifth and sixth segments. First segment 2× as long as wide; fifth segment without suture lines and with spine-like element with bifid tip; sixth segment with two subequal elements. Armature formula: 1-[1], 2-[11], 3-[8], 4-[2], 5-[11 + 1 element + (1 + ae)], 6-[2 elements], 7-[1], 8-[9 + acrothek].

P2, inner setae on exp-2 and exp-3 longer than those in females. Enp-2 (Fig. 8E) shorter than length of enp-1, with one outer apophysis and two distal setae (inner short and outer long); apophysis ~ 1.3× as long as endopod.

P3, inner setae on exp-2 and exp-3 longer than those in females. Enp-2 (Fig. 8F) with two long apical setae and slightly curved outer spine bearing one row of spinules along one margin; outer spine ~ 2/5 length of inner apical seta.

P4, exp-2 (Fig. 8G) with inner seta shorter than in females, and outer distal corner modified into elongate and recurved process with blunt tip (indicated by arrowhead in Fig. 8G); exp-3 (Fig. 8G) with six elements (two inner, two apical, and two outer). Enp-2 (Fig. 8H), inner apical seta distinctly shorter than in female, nearly half as long as outer apical seta.

P5 (Fig. 9F). Baseoendopods fused medially, each with one outer basal seta and four anterior pores; endopodal lobe small and narrow, reaching proximal 1/5 of exopod, with one long inner bipinnate spine, one distal bipinnate spine, and one distal slender seta. Exopods asymmetrical, with unequal segmentation (right leg incompletely two-segmented, with a suture visible only on anterior surface and left leg one-segmented); distal margin strongly produced into a long apical pinnate process; with one inner pinnate seta, four naked, unequal outer setae (first proximal seta longest, second proximal seta shortest), and with two pores on anterior surface.

P6 (Fig. 9D) asymmetrical as in *T. concavus* sp. nov., distally with one spine and two setae, middle seta 2× as long as innermost spine.

#### Etymology.

The specific epithet *spinulifer* is derived from the Latin *spinula* (small spine) and *-fer* (bearing), and refers to the presence of numerous spinules on the female caudal rami and on the apical process of the male fifth leg exopod. It is in the nominative singular. Gender masculine.

#### Variability.

The P5 were asymmetrical (Fig. 9F) in all male specimens examined (*n* = 4), with a one-segmented exopod on one side and an incompletely two-segmented exopod on the other.

#### Remarks.

*Tetragoniceps spinulifer* sp. nov. shares the same armature formulae of P1–P4 with *Tetragoniceps concavus* sp. nov., except for the presence of only two setae on P1enp-2. The female caudal ramus is relatively short, being only 1.6× as long as wide, a condition shared only with *T. brevicauda*, *T. prima*, and *T. truncata* among known *Tetragoniceps* species (Scott 1900; Nicholls 1939; Coull 1971). However, the new species differs conspicuously from the latter three species in the shape of the caudal ramus, which tapers distinctly along the posterior two-fifths in lateral view and bears long spinules along the inner margin and ventroposterior surface. Among the five newly described species, the male of *T. spinulifer* sp. nov. exhibits two characteristic features: the outer distal corner of P4exp-2 is modified into an elongate, recurved process, and the P5 exopod is distally produced into a long, pinnate apical process. The male P5 exopod appears to be two-segmented because a residual suture is retained on one side; however, this condition is interpreted as incomplete fusion rather than true segmentation. This incomplete fusion differs from the condition observed in *T. bergensis*, *T. prima*, and *T. santacruzensis*, in which the P5 exopod is clearly two-segmented and the distal segment bears an apical seta (Por 1965; Coull 1971; Mielke 1997).

### Tetragoniceps brevisetosus sp. nov.

Taxon classificationAnimaliaHarpacticoidaTetragonicipitidae

48C2DEE8-211B-5A80-A63D-047C1363E50F

https://zoobank.org/3AC014B2-02BC-4E6B-9A1F-1BE875753CED

Figs 10, 11, 12, 13

#### Type locality.

Dokdo Island, Korea; 37°14'59.23"N, 131°52'23.28"E; depth 66.5 m.

#### Type material.

***Holotype***. • One ♀ (MABIK CR00261571) preserved in a vial with 80% ethanol; collected from the type locality, 02 November 2018, J.G. Kim leg. ***Allotype***. • One ♂ (MABIK CR00261572) preserved in a vial with 80% ethanol, collection data as in holotype. ***Paratypes***. • One ♀ (MABIK CR00261573) undissected on a H-S slide; • two ♀♀ (MABIK CR00261574, MABIK CR00261575) and two ♂♂ (MABIK CR00261576, MABIK CR00261577) each dissected on three H-S slides, collection data as in holotype.

#### Description.

**Figure 10.**
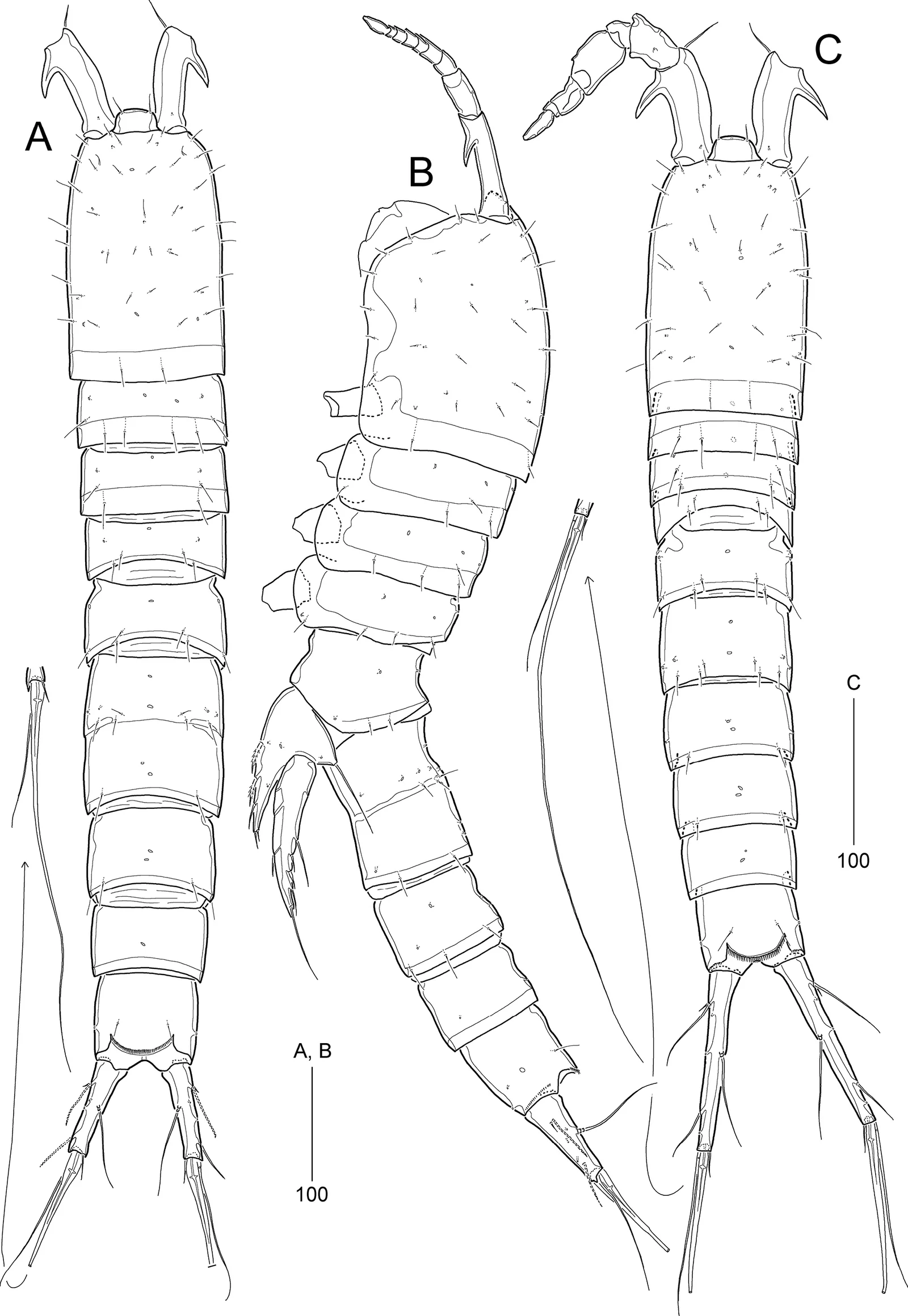
*Tetragoniceps brevisetosus* sp. nov. Paratype (MABIK CR00261573), female. **A**. Habitus, dorsal; **B**. Habitus, lateral. Allotype (MABIK CR00261572), male; **C**. Habitus, dorsal. Scale bars are in μm.

**Female**. Body length range 906–968 μm (*n* = 4, mean = 934 μm, holotype = 906 μm). Habitus (Fig. 10A, B) subcylindrical, without clear demarcation between prosome and urosome; surface covered with small pits (not figured), more conspicuous than in *T. concavus* sp. nov. Cephalothorax (Fig. 10A) ~ 1.6× as long as wide, greatest width 138 μm measured at posterior end of cephalothorax.

Urosome (Fig. 10A, B) five-segmented, ornamentation as figured. Genital double-somite separated by faint dorsal line and lateral chitinous ribs, but completely fused ventrally. Copulatory pore (Fig. 12F) located at approximately midpoint of double-somite length. P6 (Fig. 12F) with three setae; innermost seta longest and naked, middle seta ~ 1/5 length of innermost seta, and outermost seta plumose and slightly shorter than inner seta. Anal somite (Figs 10A, 10B, 12G) slightly longer than penultimate somite, with one pair of dorsal sensilla, one pair of lateral pores, and three pairs of ventral pores; anal operculum semicircular, wide, ornamented with minute spinules along posterior margin.

Caudal rami (Figs 10A, 10B, 12G) slightly divergent, ~ 4.1× as long as wide, as long as penultimate somite; each ramus with three pores, a minute ventroposterior protuberance, and seven setae: setae I and II positioned on anterior 1/4 of outer margin; seta I diminutive; seta II slender and naked, ~ 1/2 length of ramus; seta III arising from anterior 4/5 of outer margin, slightly shorter than seta II; setae IV and V fused basally; seta IV ~ 1.5× as long as ramus; seta V longest; seta VI short, ~ 1/2 length of seta II; seta VII tri-articulated, inserted dorsally in mid-length of ramus, slightly longer than ramus.

Rostrum (Fig. 10A) distinct, with two subapical sensilla and one apical median pore.

**Figure 11.**
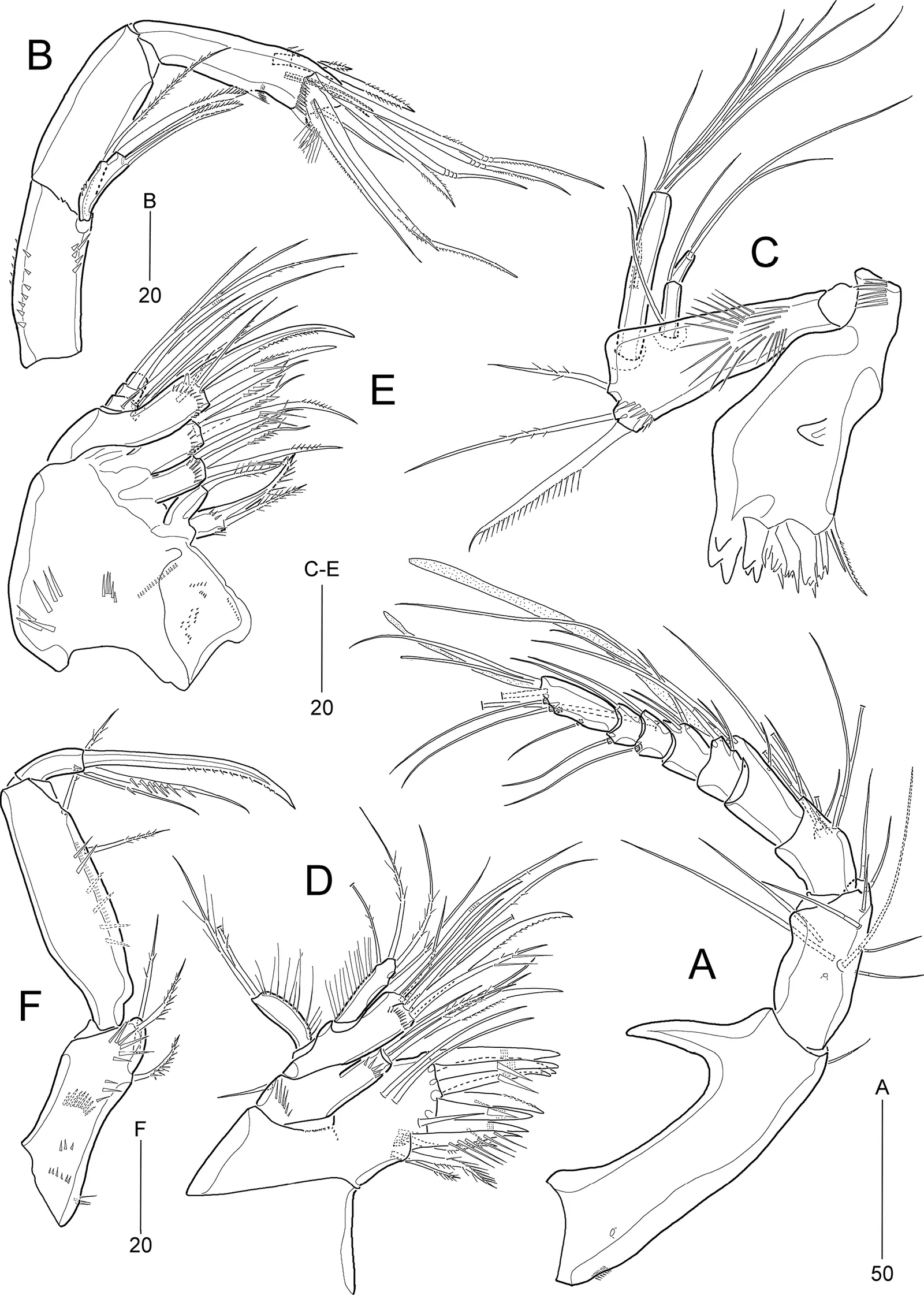
*Tetragoniceps brevisetosus* sp. nov. Paratype (MABIK CR00261574), female. **A**. Antennule; **B**. Antenna; **C**. Mandible; **D**. Maxillule; **E**. Maxilla; **F**. Maxilliped. Scale bars are in μm.

Segmentation and setal armature of cephalothoracic appendages (Fig. 11A–F) as in *T. concavus* sp. nov. apart from the following features: first segment of antennule ~ 2× as long as second segment (Fig. 11A); antennary (Fig. 11B) basis with three rows of spinules, exopod with only few spinules, inner distal seta on exopod slightly longer than outer distal seta, and distal endopodal segment lacking inner proximal ornamentation; mandibular exopod elongate, exceeding mid-length of endopod, proximal segment 1.2× as long as distal segment, and distal exopodal segment with two apical setae (Fig. 11C); maxillule with one naked epipodal seta on coxa, and two apical setae on exopod (Fig. 11D); maxillipedal endopod with one subdistal tube pore, two unipinnate setae, and distal claw (Fig. 11F).

**Figure 12.**
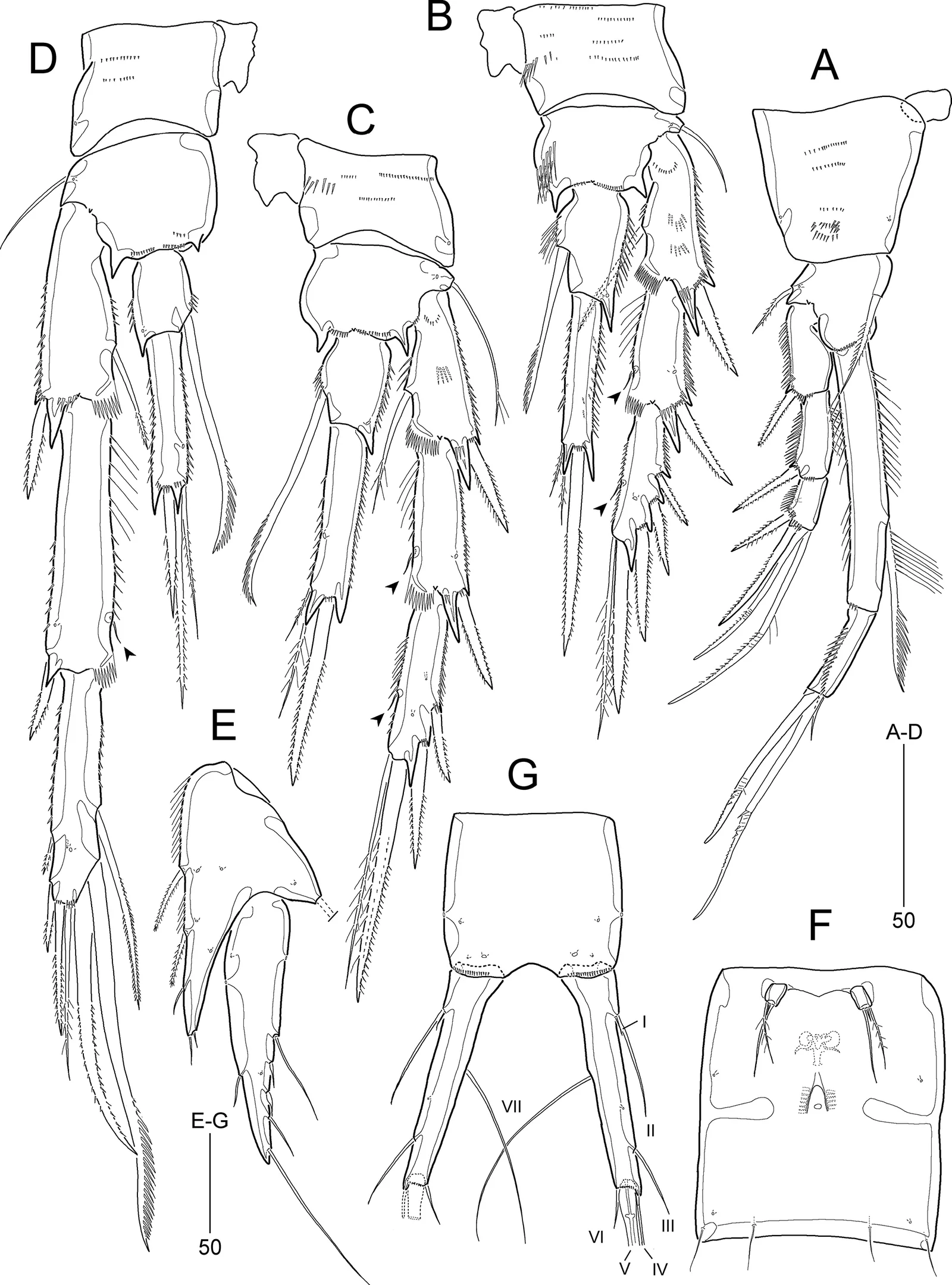
*Tetragoniceps brevisetosus* sp. nov. Paratype (MABIK CR00261574), female. **A**. P1, anterior; **B**. P2, anterior; **C**. P3, anterior; **D**. P4, anterior; **E**. P5, anterior; **F**. Genital double-somite, ventral; **G**. Anal somite and caudal rami, ventral. Scale bars are in μm.

Segmentation and setation of P1–P4 as in *T. concavus* sp. nov. (Fig. 12A–D) apart from the following features; P1enp-1 elongate, distinctly longer than exopod, ~ 2.7× as long as enp-2, inner seta of enp-1 reaching slightly beyond mid-length of enp-2, and enp-2 4× as long as wide; inner setae on P2–P4exp-2 and P2–P3exp-3 delicate, difficult to discern; outer spines on P2–P3enp-2 longer than supporting segments and apical setae short; outer spine on P4enp-2 shorter than supporting segment, outer apical seta longest, ~ 1.2× as long as inner apical seta.

P5 (Fig. 12E). Baseoendopod gradually broadening distally, ornamented with inner setules and five anterior pores; triangular endopodal lobe exceeding mid-length of exopod, with one naked seta (subdistal) and two pinnate setae (proximal) on inner margin, and two small naked setae at apical tip. Exopod slenderer than in *T. concavus* sp. nov., tapering distally to pointed tip, ~ 4.3× as long as wide; with one short inner seta and five outer setae; second and third proximal outer setae short and bulbous at base, and subdistal seta longest, ~ 0.6× as long as exopod; with four pores (three proximal and one distal).

**Male**. Body length range 660–745 μm (*n* = 3, mean = 695 μm). Habitus (Fig. 10C) as in females, but with sexual dimorphism in antennule, segmentation of genital somite, caudal rami, and P2–P6.

**Figure 13.**
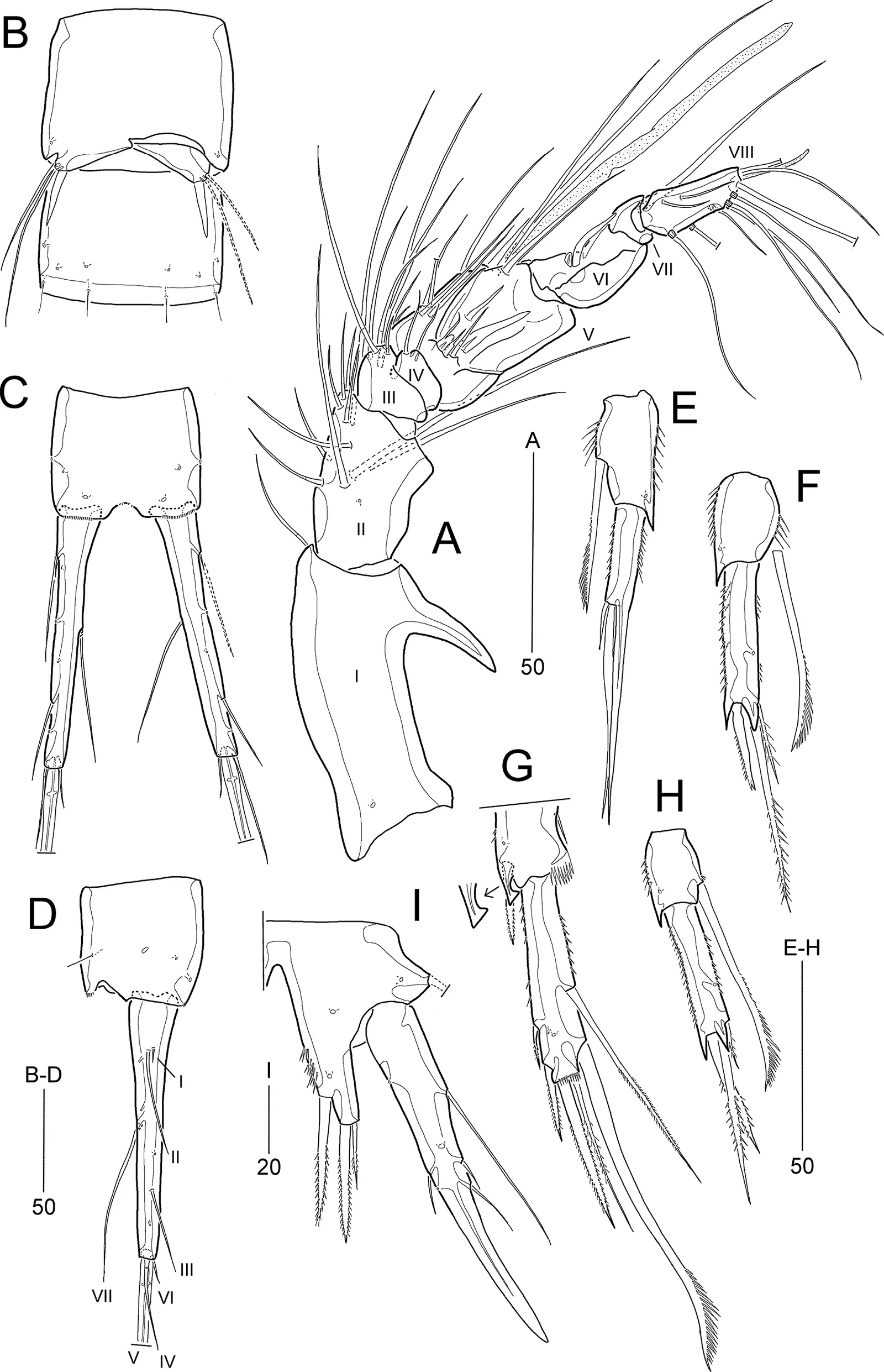
*Tetragoniceps brevisetosus* sp. nov. Paratype (MABIK CR00261576), male. **A**. Antennule; **B**. Genital somite and first abdominal somite, ventral; **C**. Anal somite and caudal rami, ventral; **D**. Anal somite and caudal ramus, lateral; **E**. P2 endopod, anterior; **F**. P3 endopod, anterior (inner seta based on another paratype); **G**. P4 exopod, distal part of second segment and third segment, anterior; **H**. P4 endopod; **I**. P5, anterior. Scale bars are in μm.

Genital somite and first abdominal somite completely separate (Figs 10C, 13B).

Caudal rami (Figs 10C, 13C, 13D) divergent, cylindrical, distinctly longer than those of female, ~ 2× as long as penultimate somite, ~ 6× as long as wide, with seven setae: seta III arising from anterior 3/4 of lateral margin; seta VII (Figs 10C, 13C, 13D) inserted at 1/2 of ramus dorsally.

Antennule (Fig. 13A) eight-segmented, subchirocer. Fifth segment swollen, with partial suture on outer surface. Armature formula as in *T. concavus* sp. nov., except for a modified proximal seta on the fifth segment, composed of a blunt proximal part and a setiform distal part.

P2 enp-2 (Fig. 13E) as long as enp-1, with two distal setae (inner short and outer long) and one outer apophysis, 2.2× as long as enp-2.

P3, outer spine on enp-2 (Fig. 13F) short and modified (bearing minute spinules unilaterally, with bifid tip), ~ 1/2 length of enp-2, outer distal seta distal half bipinnate, ~ 2.3× as long as outer spine.

P4exp-2 (Fig. 13G) with modified outer distal corner with concave inner margin and blunt tip (shown separately in Fig. 13G). Exp-3 (Fig. 13G) with six elements (two inner, two apical, and two outer). Inner distal seta on enp-2 (Fig. 13H) short, 1/4 length of adjacent distal seta.

P5 (Fig. 13I) with baseoendopods fused medially, each with one outer basal seta (damaged) and three anterior pores; endopodal lobe with spinules on inner margin subdistally; with one inner bipinnate spine, one distal bipinnate spine, and one distal pinnate seta; outer distal seta ~ 1/2 length of inner distal spine. Exopod one-segmented, elongate, ~ 6.8× as long as wide, distally produced into a long apical process occupying 40% of exopod; with one short inner seta, one long (proximal) and two short (subdistal) outer setae, and three pores.

P6 (Fig. 13B) asymmetrical as in *T. concavus* sp. nov., each small plate with one short spine and two slender setae; outermost seta ~ 2/3 length of adjacent inner seta.

#### Etymology.

The specific epithet *brevisetosus* is derived from the Latin *brevis* (short) and *setosus* (bearing setae), and refers to the markedly short inner setae on the second exopodal segment of the second to fourth swimming legs. It is in the nominative singular. Gender masculine.

#### Variability.

The P3enp-1 bears one inner seta in all examined specimens (*n* = 7), except for the figured male paratype (MABIK CR00261576; Fig. 13F), in which the inner seta is absent on the right side and is represented by a minute insertion mark on the left.

#### Remarks.

*Tetragoniceps brevisetosus* sp. nov. shares the same armature formulae of P1–P4 with *T. concavus* sp. nov., but differs in having extremely short inner setae (difficult to discern at low magnification) on P2–P3exp-2 and exp-3, and P4exp-2. The new species is further distinguished by comparatively short apical setae on P2–P4enp-2. In the female, the outer apical seta on P2–P3enp-2 is markedly shorter than the outer spine, whereas in the other Korean species it is longer than the outer spine. The inner apical seta of the female P4enp-2 is only slightly longer than the outer spine, whereas in the remaining Korean species, it is more than 1.4× as long as the outer spine. In the male, the inner apical seta of P4enp-2 is even shorter, being < 1/2 as long as the outer spine. The male shows sexual dimorphism, and is expressed in the elongate caudal rami, as in *T. spinulifer* sp. nov. However, it differs from *T. spinulifer* sp. nov. in the shape of the P5 exopod, which bears an elongate, slender distal extension and reduced armature consisting of one inner and three outer setae.

### Tetragoniceps unisetus sp. nov.

Taxon classificationAnimaliaHarpacticoidaTetragonicipitidae

DF861330-88AC-5468-8F3C-7C8EE22D2179

https://zoobank.org/289D6787-B78E-4F32-84A7-35791C3A6F52

Figs 14, 15, 16, 17

#### Type locality.

South Sea of Korea; 33°40'22.57"N, 127°30'01.02"E; depth 93.7 m.

#### Type material.

***Holotype***. • One ♀ (MABIK CR00261578) preserved in a vial with 80% ethanol; collected from the type locality, 29 November 2016, S.L. Kim leg. ***Allotype***. • One ♂ (MABIK CR00261579) preserved in a vial with 80% ethanol, collection data as in holotype. ***Paratypes***. • Three ♀♀ (MABIK CR00261580–CR00261582) and two ♂♂ (MABIK CR00261583, MABIK CR00261584) each dissected on three H-S slides, collection data as in holotype.

#### Description.

**Figure 14.**
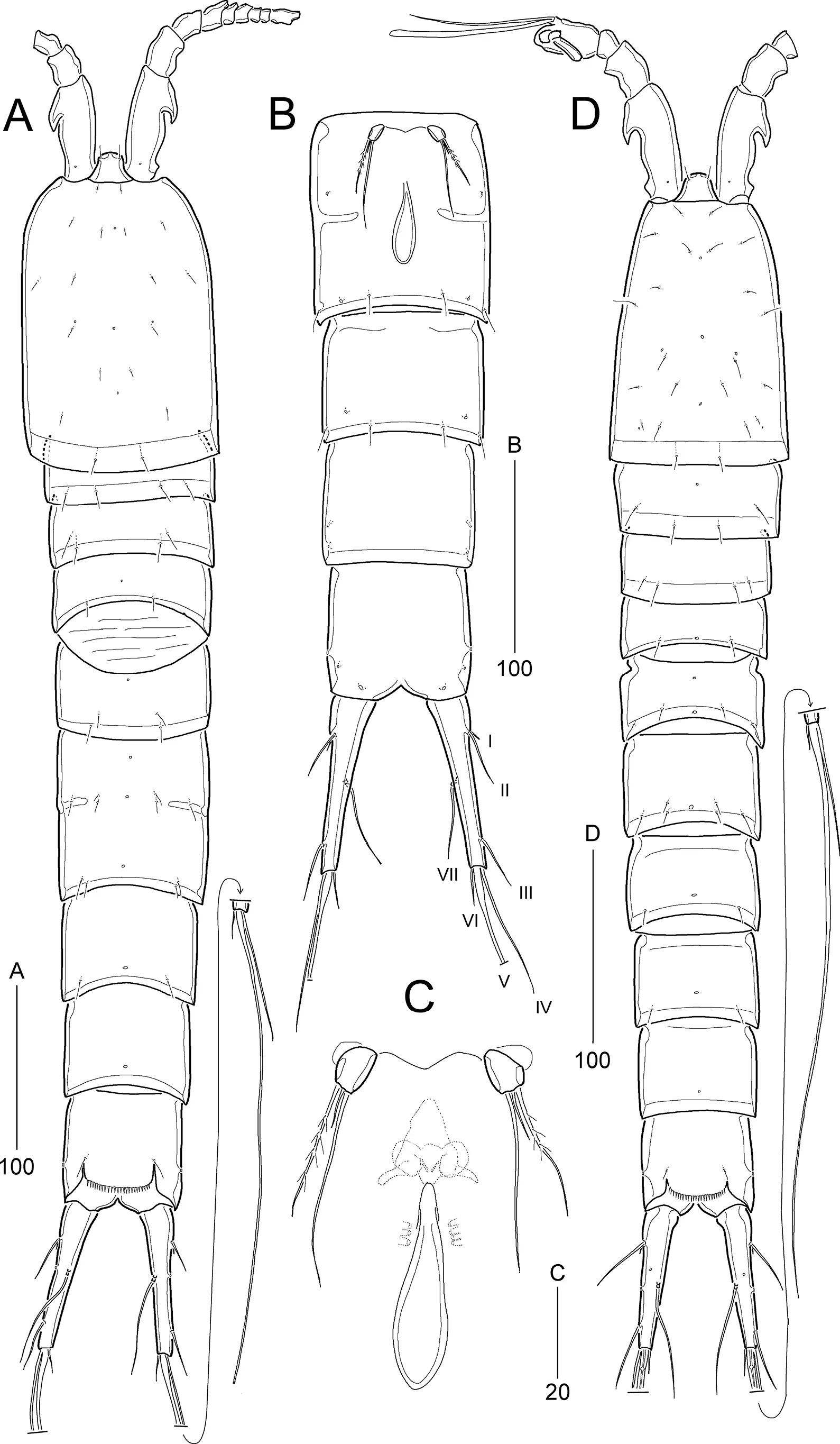
*Tetragoniceps unisetus* sp. nov. Paratype (MABIK CR00261580), female. **A**. Habitus, dorsal; **B**. Urosome, ventral; **C**. Genital field. Paratype (MABIK CR00261583), male; **D**. Habitus, dorsal. Scale bars are in μm.

**Female**. Body length 704–769 μm (*n* = 4, mean = 740 μm, holotype = 769 μm). Habitus (Fig. 14A) smaller and slenderer than in *T. concavus* sp. nov. Cephalothorax (Fig. 14A) ~ 1.5× as long as wide, greatest width 116 μm measured at posterior end of cephalothorax.

Body segmentation (Fig. 14A, B) as in *T. concavus* sp. nov., ornamentation as figured. Genital double-somite fused ventrally and dorsally, without a faint dorsal line. Each P6 (Fig. 14C) with three setae; outer plumose seta ~ 3/4 length of inner seta, and middle seta as long as P6. Anal somite (Fig. 14A, B) as long as penultimate somite.

Caudal rami (Fig. 14A, B) divergent, elongate, ~ 4.1× as long as wide, ornamented with a pair of lateral pores; each ramus with seven slender setae: setae I and II positioned at anterior 1/5 of outer margin; seta III arising from anterior 4/5 of outer margin, slightly longer than seta II; setae IV and V fused basally; seta IV ~ 3× as long as seta II; seta V longest; seta VI ~ 2/3 length of seta II; seta VII tri-articulate, inserted dorsally in mid-length of ramus, ~ 4/5 length of ramus.

Rostrum (Fig. 14A) distinct, with two sensilla on apical margin and one apical median pore; lateral margins slightly concave.

**Figure 15.**
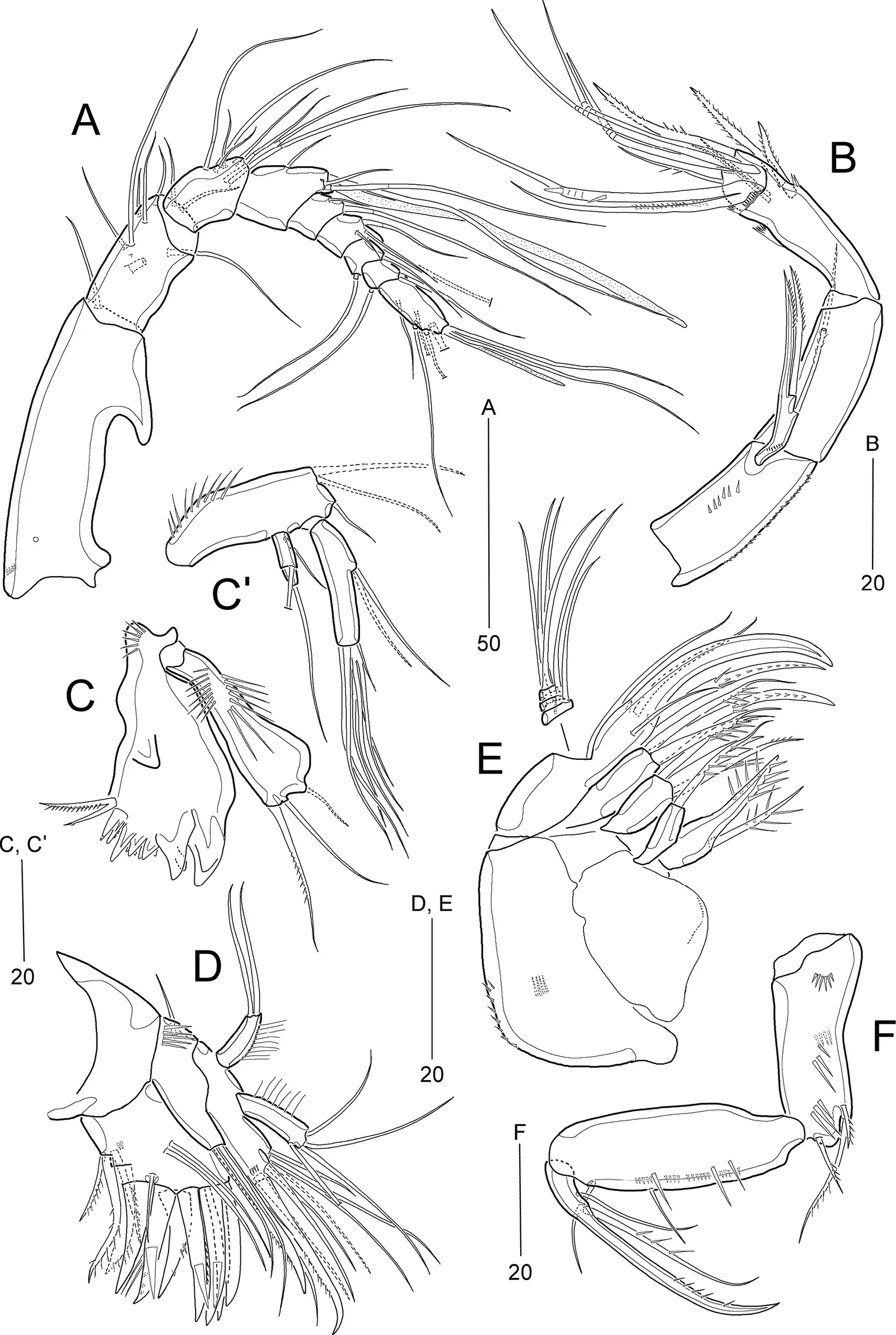
*Tetragoniceps unisetus* sp. nov. Paratype (MABIK CR00261580), female. **A**. Antennule; **B**. Antenna; **C**. Mandible; **C’**. Mandibular palp, endopod and exopod, opposite side; **D**. Maxillule; **E**. Maxilla; **F**. Maxilliped. Scale bars are in μm.

Segmentation and setal armature of cephalothoracic appendages (Fig. 15A–F) as in *T. concavus* sp. nov. apart from the following features: first segment of antennule with small and backwardly recurved process, ~ 1.8× as long as second segment (Fig. 15A); distal endopodal segment of antenna lacking tube pore on distal margin and spinular row on inner margin (Fig. 15B); basis of mandibular palp lacking distal ornamentation of spinules, with three setae, of which one seta ~ 1/2 length of other setae, and distal exopodal segment with only one apical seta (Fig. 15C); maxillular exopod with two setae (Fig. 15D); basis of maxilliped with two naked setae (Fig. 15F).

**Figure 16.**
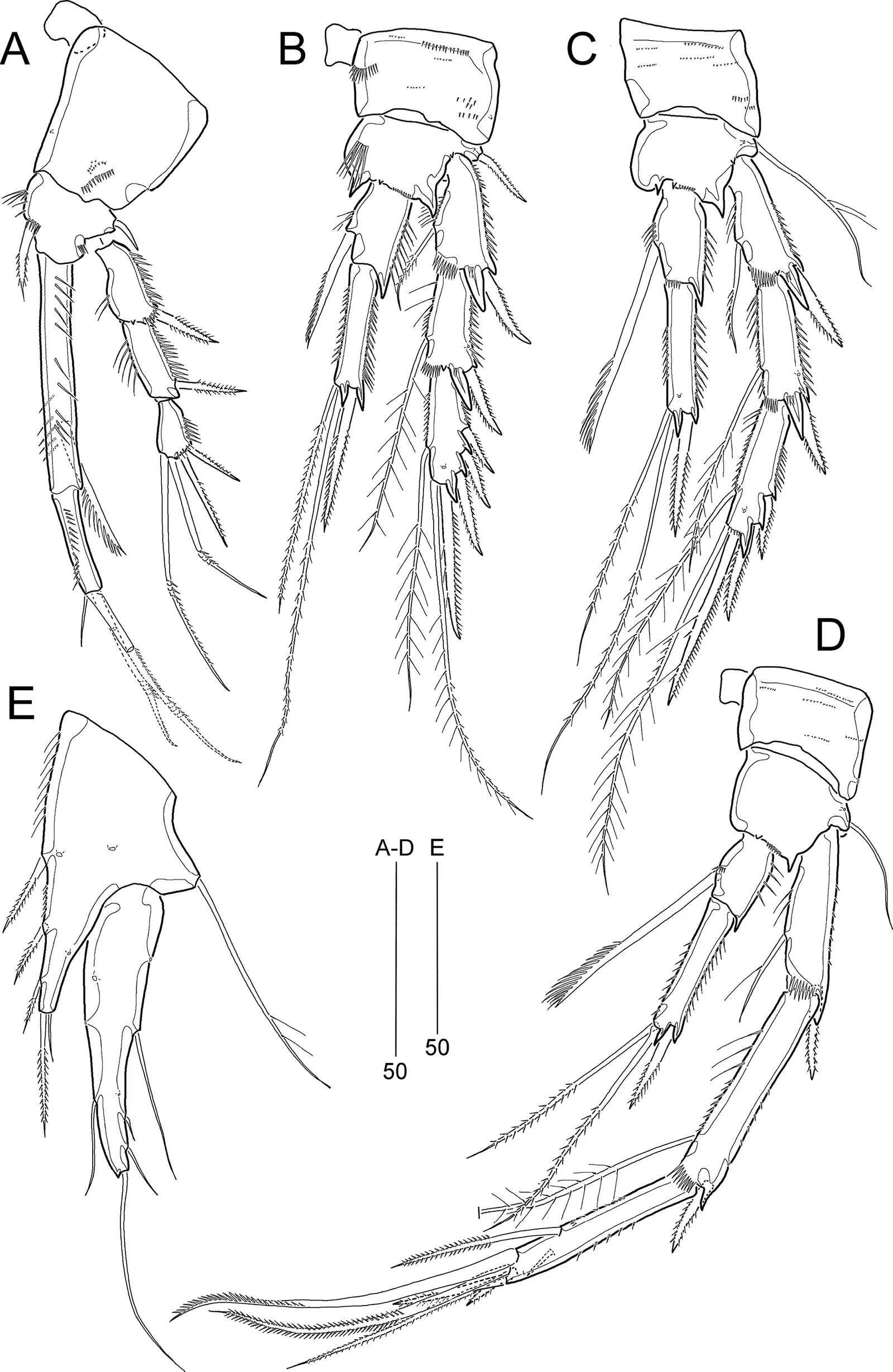
*Tetragoniceps unisetus* sp. nov. Paratype (MABIK CR00261580), female. **A**. P1, anterior; **B**. P2, anterior; **C**. P3, anterior; **D**. P4, anterior; **E**. P5, anterior. Scale bars are in μm.

Segmentation and setation of P1–P4 as in *T. concavus* sp. nov., and ornamentation as in Fig. 16A–D. Distinct differences from *T. concavus* sp. nov. in the following: P1 (Fig. 16A), enp-1 ~ 2.3× as long as enp-2, inner seta of enp-1 short, reaching proximal 2/3 of enp-2, and enp-2 ~ 4× as long as wide; outer spine on P2–P4enp-2 relatively short, ~ 2/3 length of inner apical seta in P2, ~ 1/2 length in P3, and ~ 2/5 length in P4 (Fig. 16B–D).

P5 (Fig. 16E) as in *T. concavus* sp. nov. apart from the following features: baseoendopod with three anterior pores; distal part of endopodal lobe narrowing abruptly, three setae on inner margin gradually becoming shorter towards distal end, and inner distal seta slightly shorter than length of endopodal lobe; exopod elongate, ~ 4.5× as long as wide, slightly recurved outwardly, with one inner seta, one long apical seta, and three outer setae (first proximal one ~ 1/2 length of exopod, second proximal one shortest and bulbous at base); tip of exopod blunt, with one small inner protrusion.

**Male**. Body length 606–628 μm (*n* = 3, mean = 616 μm). Habitus (Fig. 14D) as in females, but with sexual dimorphism in antennule, genital segmentation, caudal rami, and P2–P6.

Genital somite and first abdominal somite completely separate (Fig. 14D).

**Figure 17.**
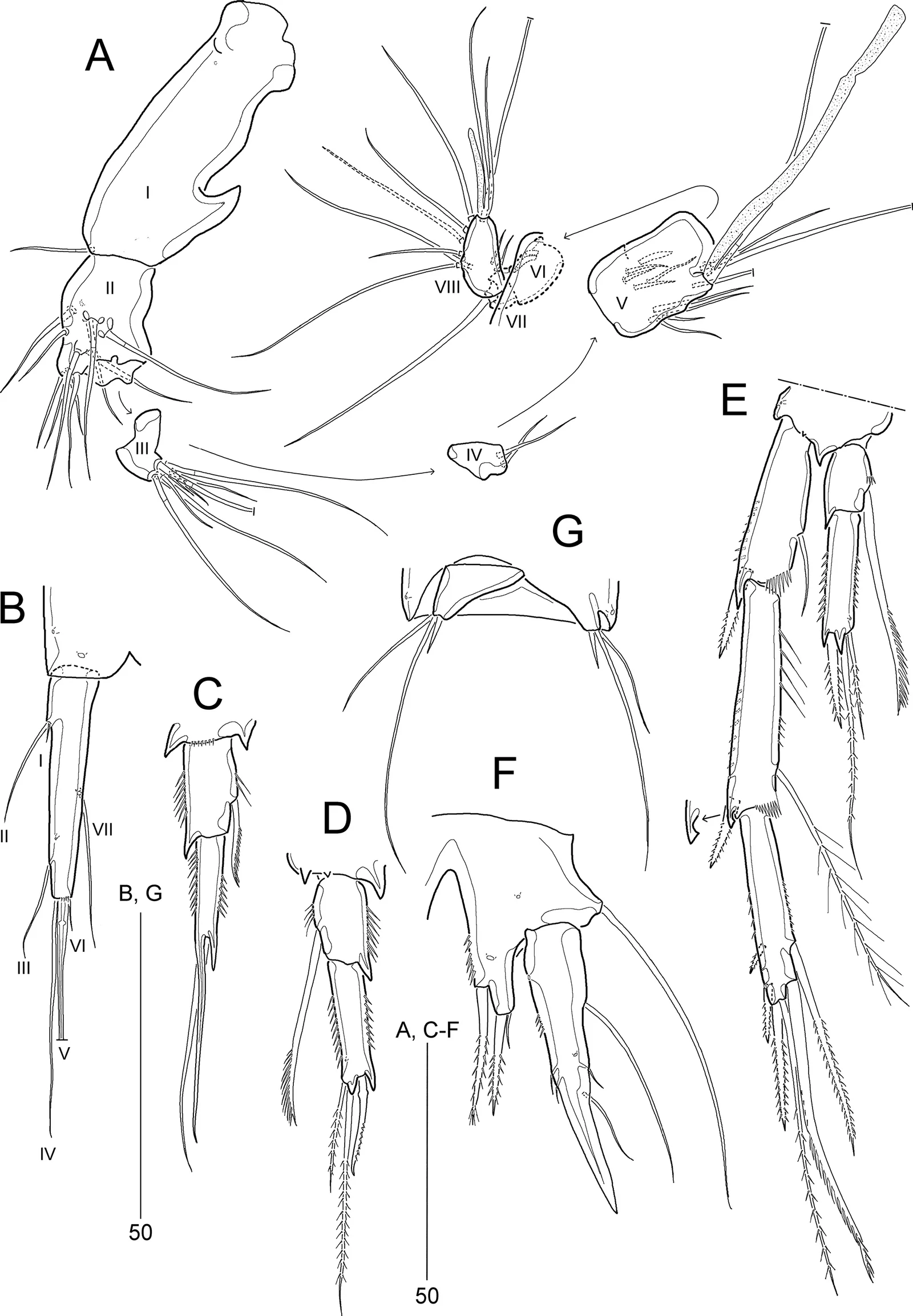
*Tetragoniceps unisetus* sp. nov. Paratype (MABIK CR00261583), male. **A**. Antennule; **B**. Caudal ramus, ventral; **C**. P2 endopod, anterior; **D**. P3 endopod, anterior; **E**. P4, anterior; **F**. P5, anterior; **G**. P6. Scale bars are in μm.

Caudal rami (Figs 14D, 17B), length-to-width ratio as in females, but additionally with one dorsal and one ventral pore on each ramus.

Antennule (Fig. 17A) eight-segmented, subchirocer. Outer process on first segment small and recurved outwardly as in female. Fifth segment slightly swollen, with partial suture line on outer surface. Armature formula as in *T. concavus* sp. nov.

P2 as in female, but inner setae on exp-2 and exp-3 longer than those in females. Enp-2 (Fig. 17C) as long as enp-1, with one long outer apophysis (2× as long as enp-2) and only one long apical naked seta (slightly longer than apophysis); inner distal corner modified into an acute process.

P3 as in female, but inner setae on exp-2 and exp-3 longer than those in females. Outer spine on enp-2 (Fig. 17D) modified, slender and bearing lateral projections proximally, and inner apical seta not exceeding half-length of outer apical seta.

P4 (Fig. 17E) as in female, but exp-2 with modified outer distal corner as in *T. concavus* sp. nov. (shown separately in Fig. 17E). Exp-3 with six elements (two inner, two apical, and two outer). Enp-2 with slightly short inner apical seta, not exceeding half-length of outer apical seta.

P5 (Fig. 17F) similar to *T. concavus* sp. nov., but outer apical seta on endopodal lobe short, naked, ~ 1/3 length of adjacent apical spine. Exopod elongate, ~ 5.4× as long as wide, with two inner setae and three outer setae; relative length and position of outer setae similar to *T. concavus* sp. nov., but two inner setae diminutive (difficult to discern, slightly shorter than middle outer seta), located close to each other; inner margin with one pore proximally and one row of spinules between pore and set of setae, anterior surface with one pore.

P6 (Fig. 17G) similar to *T. concavus* sp. nov., except innermost seta slender and short, ~ 1/4 length of outermost seta.

#### Etymology.

The specific epithet *unisetus* is derived from the Latin *unus* (one) and *seta* (bristle), and refers to the presence of a single seta on the second segment of the mandibular exopod. It is in the nominative singular. Gender masculine.

#### Variability.

The inner seta on the P2 enp-2 exceeds the outer spine in all examined females (*n* = 4), except for the figured female paratype (MABIK CR00261580), in which the inner seta on the P2 enp-2 is as long as the outer spine on one side. The P2 enp-1 bears one inner seta in all examined males (*n* = 3), except for the figured male paratype (MABIK CR00261583), with two inner setae on the P2 enp-1, asymmetrically.

#### Remarks.

*Tetragoniceps unisetus* sp. nov. is easily distinguished from the other four new species by the comparatively small process on the first antennulary segment, the presence of a single apical seta on the mandibular exopod, two setae on the maxillular exopod, and five setae on the female P5 exopod, as well as the absence of sexual dimorphism in the caudal ramus. The morphology of the female P5 exopod provides the most useful character for distinguishing the five new species. The length-to-width ratio of the female P5 exopod is lower in *T. protuberans* sp. nov. (3.1) and *T. concavus* sp. nov. (3.1) than in the remaining species (4.5 in *T. unisetus* sp. nov., 4.3 in *T. brevisetosus* sp. nov., and 5 in *T. spinulifer* sp. nov.). The apical tip is pointed in *T. concavus* sp. nov., *T. brevisetosus* sp. nov., and *T. protuberans* sp. nov., whereas it bears an apical seta in *T. spinulifer* sp. nov. and *T. unisetus* sp. nov. The male of *T. unisetus* sp. nov. exhibits two distinctive characteristics. The P2 enp-2 bears only one outer apophysis and one long apical seta. Notably, a conspicuous process occurs adjacent to the long apical seta, a feature not observed in other congeners. Given its insertion point, this process likely represents a modified inner apical seta associated with sexual dimorphism. This sexually dimorphic modification appears to be unique within the genus. Furthermore, the male P5 exopod is characterized by two remarkably short inner setae positioned close together. In *T. spinulifer* sp. nov. and *T. brevisetosus* sp. nov., only a single inner seta is present.

### Tetragoniceps protuberans sp. nov.

Taxon classificationAnimaliaHarpacticoidaTetragonicipitidae

31E97003-07D2-5C29-BD40-6DA243462DE9

https://zoobank.org/967A8C7E-F5BD-484F-AB3D-28E0231063F4

Figs 18, 19, 20, 21

#### Type locality.

Gwangyang, Korea; 34°56'10.26"N, 127°36'06.95"E; depth 0~1 m.

#### Type material.

***Holotype***. • One ♀ (HNIBRIV25103) preserved in a vial with 95% ethanol, collected from the type locality, 15 October 2016, J.G. Kim leg. ***Allotype***. • One ♂ (HNIBRIV25104) preserved in a vial with 95% ethanol, collection data as in holotype. ***Paratypes***. • Two ♀♀ (HNIBRIV25105, HNIBRIV25106) and two ♂♂ (HNIBRIV25107, HNIBRIV25108) each dissected on three H-S slides, collection data as in holotype.

#### Description.

**Figure 18.**
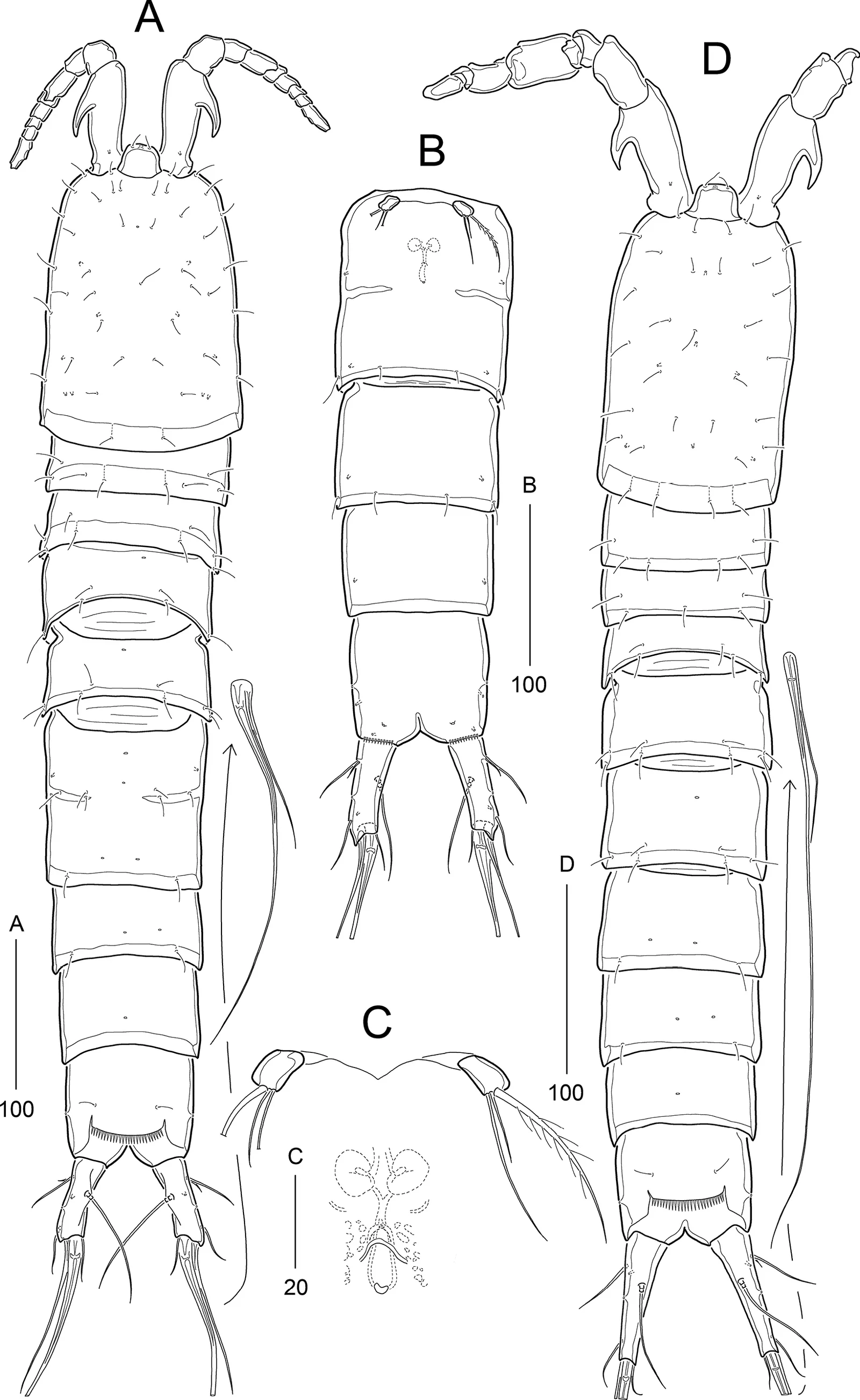
*Tetragoniceps protuberans* sp. nov. Holotype (HNIBRIV25103), female. **A**. Habitus, dorsal. Paratype (HNIBRIV25105), female; **B**. Urosome, ventral; **C**. Genital field. Allotype (HNIBRIV25104), male; **D**. Habitus, dorsal. Scale bars are in μm.

**Female**. Body length range 737–749 μm (*n* = 3, mean = 745 μm, holotype = 737 μm). Habitus (Fig. 18A) subcylindrical, without clear demarcation between prosome and urosome; cephalothorax (Fig. 18A) ~ 1.4× as long as wide, greatest width 134 μm measured at posterior end of cephalothorax.

Urosome (Fig. 18A, B) as in *T. concavus* sp. nov., ornamentation as figured. Genital double-somite separated laterally by internal chitinous ribs, but fused dorsally and ventrally, lacking a faint dorsal line; genital apertures located near anterior margin and median copulatory pore small, situated in anterior 1/3 of genital double-somite; P6 (Fig. 18C) similar to *T. concavus* sp. nov., except for naked inner seta slightly shorter than outer plumose seta and middle seta small, not exceeding mid-length of inner seta.

Caudal ramus (Figs 18A, 18B, 20F) cylindrical, slightly shorter than anal somite, ~ 3× as long as wide; ventroposterior margin with a small protuberance at outer distal corner (indicated by arrowhead in Fig. 20F), more prominent than in *T. concavus* sp. nov.; with three pores, one each on dorsal, ventral, and lateral surfaces; with seven setae: setae I and II positioned on anterior 1/4 of lateral margin; seta I diminutive; seta II 2/3 length of ramus; seta III arising from anterior 3/4 of lateral margin, slightly shorter than seta II; setae IV and V fused basally; proximal part of seta V swollen and slightly recurved outwardly; seta IV slender, approximately as long as penultimate somite and anal somite combined; seta V longest, as long as urosome excluding P5-bearing somite; seta VI small, slightly shorter than seta II; seta VII inserted at anterior 2/5 of dorsal surface.

Rostrum (Fig. 18A) similar to *T. concavus* sp. nov., with one apical median pore and two subapical sensilla.

**Figure 19.**
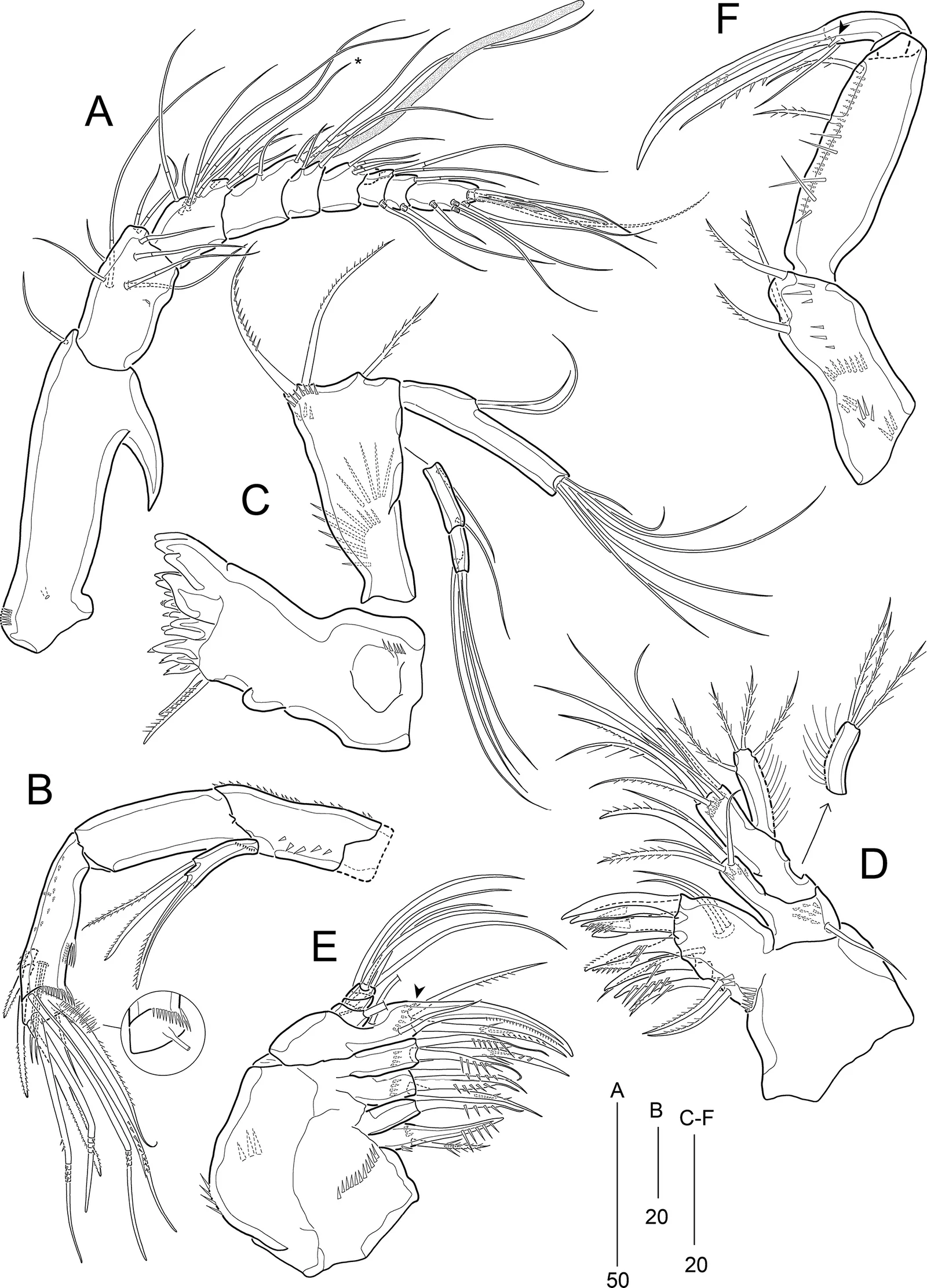
*Tetragoniceps protuberans* sp. nov. Paratype (HNIBRIV25105), female. **A**. Antennule; **B**. Antenna; **C**. Mandible; **D**. Maxillule; **E**. Maxilla; **F**. Maxilliped. Scale bars are in μm.

Segmentation and setation of cephalothoracic appendages (Fig. 19A–F) as in *T. concavus* sp. nov. apart from the following features: first segment of antennule (Fig. 19A) ~ 2× as long as second segment, with a stout outer process, and third segment with eight or nine setae, the additional seta indicated by an asterisk in Fig. 19A (see Variability); exopod of antenna (Fig. 19B) ornamented with only one row of spinules proximally; mandibular coxa with one row of spinules proximally, basis with three unequal setae (one of these distinctly shorter than other setae), and endopod with two subequal lateral setae (Fig. 19C); maxillular coxa with one naked epipodal seta, endopod slightly longer than exopod (Fig. 19D); syncoxa of maxilla with less ornamentation (one group on outer margin and two groups on anterior and posterior surfaces, respectively) (Fig. 19E); basis of maxilliped ornamented with inner spinules except for proximal 1/5, claw-like endopod ornamented with stouter spinules than in *T. concavus* sp. nov. (Fig. 19F).

**Figure 20.**
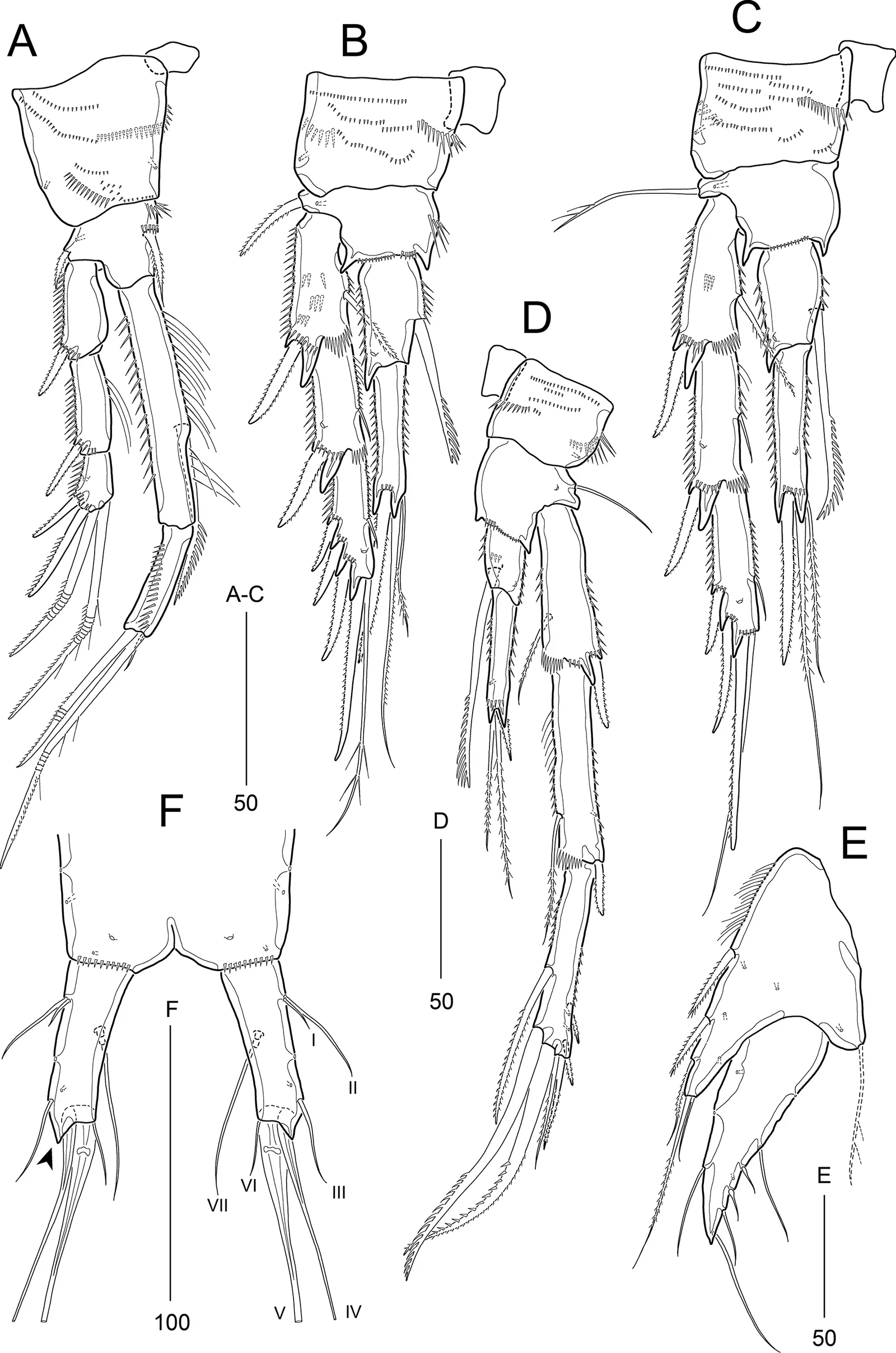
*Tetragoniceps protuberans* sp. nov. Paratype (HNIBRIV25105), female. **A**. P1, anterior; **B**. P2, anterior; **C**. P3, anterior; **D**. P4, anterior; **E**. P5, anterior; **F**. Caudal rami, ventral. Scale bars are in μm.

Segmentation and setation of P1–P4 as in *T. concavus* sp. nov. (Fig. 20A–D) apart from the following features: coxae and bases of P1–P4 with surface ornamentation as in Fig. 20A–D; P1enp-1 (Fig. 20A) slightly exceeding end of exopod, inner seta extending slightly beyond mid-length of enp-2, enp-1 ~ 2.2× as long as enp-2; P2–P3exp-2 and exp-3 (Fig. 20B, C) with short and naked inner seta, respectively, not exceeding distal margin of corresponding segment; outer spine on enp-2 subequal in length to supporting segment in P2 and P3, but only ~ 1/3 as long as in P4.

P5 (Fig. 20E) similar to *T. concavus* sp. nov. except for the following features: endopodal lobe, inner apical seta beyond tip of exopod; exopod 3.1× as long as wide.

**Male**. Body length range 618–688 μm (*n* = 3, mean = 641 μm). Habitus (Fig. 18D) as in females, except for urosomal segmentation. Sexual dimorphism observed in antennule, urosome, caudal rami, and P2–P6.

**Figure 21.**
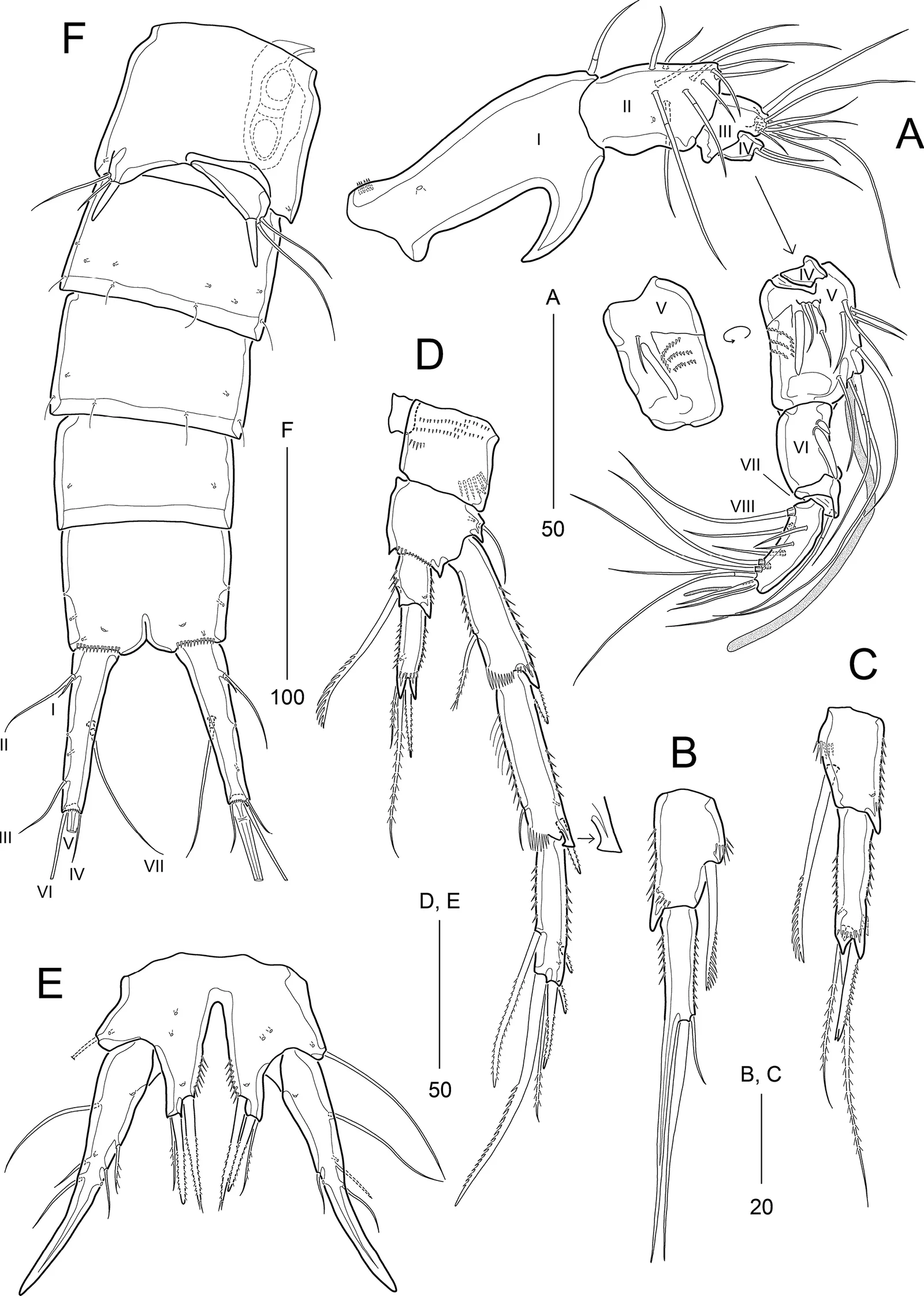
*Tetragoniceps protuberans* sp. nov. Paratype (HNIBRIV25107), male. **A**. Antennule; **B**. P2 endopod, anterior; **C**. P3 endopod, anterior; **D**. P4, anterior; **E**. P5, anterior; **F**. Urosome, ventral. Scale bars are in μm.

Urosome (Figs 18D, 21F) six-segmented, with pores and sensilla as figured; genital somite and first abdominal somite completely separated.

Caudal rami (Figs 18D, 21F) cylindrical, more distinctly tapering distally than in female, 3.1× as long as maximum width; with seven setae: seta III arising from anterior 5/6 of lateral margin; seta VII inserted at anterior 2/5 of ramus dorsally.

Antennule (Fig. 21A) eight-segmented, with same armature formula as in *T. concavus* sp. nov.; first segment 2.3× as long as wide, outer process strongly developed and recurved backwardly.

P2 enp-2 (Fig. 21B) as long as enp-1, with one apophysis and two naked setae; apophysis and outer apical seta subequal in length, ~ 2.1× as long as enp-2, and inner apical seta short, 2/3 length of enp-2.

P3enp-2 (Fig. 21C) with relatively short outer spine bearing unilateral spinules and with bifid tip, distinctly shorter than enp-2; outer spine ~ 2/3 length of inner apical seta.

P4exp-2 (Fig. 21D) with one short and naked inner seta, and outer distal corner modified as in *T. concavus* sp. nov. (shown separately in Fig. 21D); exp-3 with six elements (two inner, two apical, and two outer).

P5 (Fig. 21E) similar to *T. concavus* sp. nov., except for the presence of four anterior pores on baseoendopod. Exopod ~ 6× as long as maximum width and with one marginal and two anterior pores; spinous distal extension slender, elongate, and occupying 45% of exopod length.

P6 (Fig. 21F) as described for *T. concavus* sp. nov.

#### Etymology.

The specific epithet *protuberans* is derived from the Latin *protuberans*, meaning “protruding”, and refers to the conspicuous protuberance on the outer distal corner of the female caudal ramus. It is in the nominative singular. Gender masculine.

#### Variability.

The third segment of the antennule bears eight setae in all examined females (*n* = 2), except for the figured female paratype (HNIBRIV25105), with both third antennulary segments bearing nine setae (the additional seta indicated by an asterisk in Fig. 19A). In all examined males (*n* = 3), caudal seta III arises from the posterior 1/5 of the lateral margin of the caudal ramus and the P5 exopod bears two inner setae. However, the figured male paratype (HNIBRIV25107) differs, with caudal seta III of the left caudal ramus (Fig. 21F) inserted at the distal end and the left P5 exopod (Fig. 21E) bearing only one inner seta, resulting in asymmetrical P5 setation.

#### Remarks.

*Tetragoniceps protuberans* sp. nov. shares the short inner setae on P2–P3exp-2 and exp-3 with *T. brevisetosus* sp. nov., but it is readily distinguished from that species by the presence of a well-developed inner seta on P4exp-2 and by its unique caudal ramus morphology. The female caudal ramus is cylindrical and bears a conspicuous ventroposterior protuberance on the outer distal corner. The principal setae IV and V are proximally swollen and curved outward. Furthermore, the male caudal ramus is the shortest among the five new species, being only 3.1× as long as wide. Unlike the other four new species, *T. protuberans* sp. nov. was collected from a brackish habitat where the river meets the sea.

##### Updated key to the species of *Tetragoniceps*

All character states are based on the original descriptions and illustrations. All P1–P4 armature formulae are based on females, except for *T. longicaudata* and *T. santacruzensis*, for which male specimens were used.

**Table:** 

1	P4exp-1 with inner seta	**2**
–	P4exp-1 without inner seta	**17**
2	P4exp-2 with inner seta	**3**
–	P4exp-2 without inner seta	**14**
3	P4enp-2 with two setae/spines	***T. dubia* Thompson IC & Scott A, 1903**
–	P4enp-2 with three or four setae/spines	**4**
4	P4enp-2 with three setae/spines	**5**
–	P4enp-2 with four setae/spines	**12**
5	P3exp-2 with inner seta	**6**
–	P3exp-2 without inner seta	**16**
6	Mandibular exopod one-segmented, P4exp-3 with six setae/spines	***T. scotti* Sars GO, 1911**
–	Mandibular exopod two-segmented, P4exp-3 with seven setae/spines	**7**
7	P3exp-3 with five setae/spines	**8**
–	P3exp-3 with six setae/spines	***T. pacificus* Burgess, 1998**
8	Female P5 exopod with five setae	***T. unisetus* sp. nov**.
–	Female P5 exopod with six setae	**9**
9	P1enp-2 with two setae, female caudal rami relatively short, length/width ratio less than 2.0	***T. spinulifer* sp. nov**.
–	P1enp-2 with three setae, female caudal rami elongate, length/width ratio over 2.5	**10**
10	Mandibular exopod with two setae on distal segment, male P5 exopod with four setae	***T. brevisetosus* sp. nov**.
–	Mandibular exopod with four setae on distal segment, male P5 exopod with five setae	**11**
11	Inner seta on P2–P3exp-2 and exp-3 extending beyond distal margin of each segment	***T. concavus* sp. nov**.
–	Inner seta on P2–P3exp-2 and exp-3 not extending beyond distal margin of each segment	***T. protuberans* sp. nov**.
12	P2 exp-1 with inner seta	**13**
–	P2 exp-1 without inner seta	***T. prima* (Coull, 1971)**
13	P2 enp-2 with two setae/spines, antennule with outer process in males	***T. arenicolous* Krishnaswamy, 1957**
–	P2 enp-2 with four setae/spines, antennule without outer process in males	***T. santacruzensis* Mielke, 1997**
14	P2 enp-2 with two setae/spines	***T. brownei* Wells, 1967**
–	P2 enp-2 with three setae/spines	**15**
15	P2 exp-3 with four setae/spines, P3exp-3 with three setae/spines, female P5 baseoendopod with four setae	***T. unguis* Wells & Rao, 1987**
–	P2 exp-3 with five setae/spines, P3exp-3 with four setae/spines, female P5 baseoendopod with five setae	***T. galapagoensis* Mielke, 1989**
16	Antennary exopod with three setae, female P5 exopod (immature) with five setae	***T. longicaudata* Nicholls, 1939**
–	Antennary exopod with four setae, female P5 exopod with six setae	***T. truncata* Nicholls, 1939**
17	Female antennule eight-segmented, female P5 baseoendopod and exopod confluent	**18**
–	Female antennule nine-segmented, female P5 baseoendopod and exopod distinct	**19**
18	Antennary exopod with two setae, P3exp-3 and P4exp-3 with five, six setae/spines, respectively	***T. bookhouti* Coull, 1971**
–	Antennary exopod with three setae, P3exp-3 and P4exp-3 with four, five setae/spines, respectively	***T. malleolatus* Brady, 1880**
19	Female caudal rami short, 1.5× as long as wide	***T. brevicauda* Scott T, 1900**
–	Female caudal rami remarkably elongate, ten × as long as wide	**20**
20	P1enp-2 with three setae, P2 exp-3 and P3exp-3 with six, five setae/spines, respectively	***T. bergensis* Por, 1965**
–	P1enp-2 with two setae, P2 exp-3 and P3exp-3 with seven setae/spines, respectively	***T. bermudensis* Mussini et al., 2025**

## Discussion

The five newly described Korean species are assigned to *Tetragoniceps* based on an elongate first antennulary segment bearing a posteriorly directed acute process at the outer distal corner and a non-foliaceous female P5. Notably, all five new species share the same armature pattern of P2–P4, with one inner seta on exp-1 and exp-2 and six, five, and seven elements on exp-3 of P2–P4, respectively. No previously described *Tetragoniceps* species exhibits this combination of chaetotaxic characters (Table 1).

**Table 1.** Comparison of morphological characters among species of the genus *Tetragoniceps* [Abbreviations: A1, antennule; A2, antenna; benp, baseoendopod; CR, caudal ramus; enp-2, second endopodal segment; exp, exopod; Md, mandible; P1–P5, first to fifth legs].

**Morphological Characters**	**No. of A1 segments (**♀)	**Position of process on A1 (**♀)	**No. of setae on A2exp**	**No. of Mdexp segments**	**No. of setae on distal segment of Mdexp**	**Length-to-width ratio of CR (**♀**/**♂)	**P1**	**P2**		**P3**		**P4**		**P5 (**♀)		**P5 (**♂)			**References**
							**No. of setae on enp-2**	**Armature formula of exp**	**No. of setae on enp-2**	**Armature formula of exp**	**No. of setae on enp-2**	**Armature formula of exp**	**No. of setae on enp-2**	**benp & exp**	**No. of setae on benp / exp**	**No. of exp segments**	**distal margin of exp**	**No. of setae on benp / exp**	
Species																			
*T. malleolatus* Brady, 1880	8	distal	3	1	2	3.3:1 / -	2	-	-	0.1.022	4 ^a)^	0.1.311	3	Confluent	4 / 1 or 5 / 4 ^b)^	1 ^c)^	elongated process ^c)^	3 / 2 ^c)^	Brady 1880; Farran 1913; Monard 1935
*T. dubia* Thompson IC & Scott A, 1903	8	distal	3	-	-	2:1 / -	2	-	-	-	-	1.1.322	2	Confluent ^d)^	5 / 4	-	-	-	Thompson and Scott 1903
*T. brevicauda* Scott T, 1900	9	distal	3	-	-	1.5:1 / -	2	-	-	-	-	0.1.311 ^e)^	-	Distinct	5 / 6	-	-	-	Scott 1900
*T. scotti* Sars GO, 1911	9	distal	3	1	3	3:1 / -	3	-	-	1.1.122	3	1.1.312 or 1.1.322 ^f)^	3	Distinct	4 / 6	1	elongated process	3 / 5	Sars 1911; Scott 1900
*T. truncata* Nicholls, 1939	9	distal	4	2	4	1:1 / -	3	1.0.023	3	1.0.022 ^g)^	3 ^g)^	1.1.322	3	Distinct	5 / 6	1 ^g)^	elongated process ^g)^	3 / 5 ^g)^	Nicholls 1939; Kornev and Chertoprud 2008
*T. longicaudata* Nicholls, 1939	9	distal	3 ^♂^	2 ^♂^	5 ^♂^	2.3:1 / 5:1	2	1.0.023 ^♂^	3 ^♂^	1.0.022 ^♂^	-	1.1.322 1.1.222 ^♂^	3	Confluent ^h)^	5 / 5	1	elongated process	3 / 5	Nicholls 1939
							3 ^♂^												
*T. arenicolous* Krishnaswamy, 1957	8	distal 2/3	2	-	-	3:1 / -	2	1.1.023	2	-.1.023	-	1.1.321	4	Distinct	4 / 3	-	-	-	Krishnaswamy 1957
*T. bergensis* Por, 1965	9	distal	3	1	2	10:1 / -	3	0.1.123	3	0.1.122	3	0.1.322	3	Distinct	5 / 5	2	apical seta	3 / 5 (1.4)	Por 1965
*T. brownei* Wells, 1967	8	distal	3	1	2	2:1 / 3:1	2	1.0.022	2	1.0.021	2	1.0.311	3	Distinct	5 / 4	-	-	-	Wells 1967
*T. prima* (Coull, 1971)	8	distal 1/2	3	1	2	1:1 / 4:1	2	0.1.123	4	0.1.223	4	1.1.322	4	Distinct	5 / 4	2	apical seta	3 / 6 (1.5)	Coull 1971
*T. bookhouti* Coull, 1971	8	distal	2	1	3	4:1 / -	2	0.1.122	3	0.1.122	3	0.1.321 ^i)^	3	Confluent	5 ^j)^ / 4	1	elongated process	3 / 3	Coull 1971
*T. unguis* Wells & Rao, 1987	8	distal	3	-	-	3:1 / 3:1	2	1.0.022	3	1.0.021	3	1.0.311	3	Distinct	4 / 4	1	elongated process	3 / 3	Wells and Rao 1987
													2 ^♂^						
*T. galapagoensis* Mielke, 1989	8	distal	3	1	3	3:1 / 4:1	2	1.0.023	3	1.0.022	3	1.0.321 1.0.211 ^♂^	3	Distinct	5 / 4	1	elongated process	3 / 3	Mielke 1989
													2 ^♂^						
*T. santacruzensis* Mielke, 1997	-	-	3 ^♂^	2 ^♂^	3 ^♂^	- / 1.8:1	2 ^♂^	1.1.023 ^♂^	4 ^♂^	1.1.022 ^♂^	4 ^♂^	1.1.222 ^♂^	4 ^♂^	-	-	2 ^k)^	apical seta	3 / 6 (1.5)	Mielke 1997
*T. pacificus* Burgess, 1998	9	distal	3	2	2	2.6:1 / -	2	1.1.123	3	1.1.222	3	1.1.322	3	Distinct	5 / 6	-	-	-	Burgess 1998
*T. bermudensis* Mussini et al., 2025	9	distal	3	1	3	10:1 / -	2	0.1.223	3	0.1.223	3	0.1.322 ^l)^	3	Distinct	5 / 6	-	-	-	Mussini et al. 2025
*T. concavus* sp. nov.	9	distal	3	2	4	2.6:1 / 6.5:1	3	1.1.123	3	1.1.122	3	1.1.322 1.1.222 ^♂^	3	Distinct	5 / 6	1	elongated process	3 / 5	This study
*T. spinulifer* sp. nov.	9	distal	3	2	2	1.6:1 / 3.5:1	2	1.1.123	3	1.1.122	3	1.1.322 1.1.222 ^♂^	3	Distinct	5 / 6	1	elongated process	3 / 5	This study
*T. brevisetosus* sp. nov.	9	distal	3	2	2	4.1:1 / 6:1	3	1.1.123	3	1.1.122	3	1.1.322 1.1.222 ^♂^	3	Distinct	5 / 6	1	elongated process	3 / 4	This study
*T. unisetus* sp. nov.	9	distal	3	2	1	4.1:1 / 4.1:1	3	1.1.123	3	1.1.122	3	1.1.322 1.1.222 ^♂^	3	Distinct	5 / 5	1	elongated process	3 / 5	This study
*T. protuberans* sp. nov.	9	distal	3	2	4	3:1 / 3.1:1	3	1.1.123	3	1.1.122	3	1.1.322 1.1.222 ^♂^	3	Distinct	5 / 6	1	elongated process	3 / 5	This study

Note. All data are based on the original descriptions and illustrations, and all character states refer to adult females, unless otherwise indicated.^ a)^ The original illustration of Brady (1880) depicts the P3 endopod as one-segmented. ^b)^ The number of setae on the female P5 basoendopod and exopod follows Monard (1935). ^c)^ The data for the male P5 follow Farran (1913). ^d)^Thompson and Scott (1903) noted that the female type specimen was possibly immature; the reported P5 character state is retained here. ^e)^ The number of inner setae on the P4 exopod was inferred from the lateral habitus illustration (pl. XIV, fig. 18) in Scott (1900). ^f)^ The armature of the P4 exopod follows *T. malleolata* sensu Scott (1900), regarded as a junior synonym of *T. scotti* by Lang (1948). ^g)^ The data presented here follow Kornev and Chertoprud (2008). ^h)^Nicholls (1939) described the female type specimen as malformed; its reported P5 character state is retained here. ^i)^ The illustration (Coull 1971: fig. XI. 94) shows an armature formula of 321, whereas the accompanying text indicates 122; the former is followed here. ^j)^ The original description indicates four setae, but the illustration (fig. XI. 95) shows five; the latter is followed here. ^k)^ The segmentation of the male P5 exopod is based on the distinctly separated left exopod; the corresponding right exopod was described as only partly separated (Mielke 1997). ^l)^ The tabulated armature formula is given as 321, whereas both the original description and illustration show 322; the latter is followed here (Mussini et al. 2025). Symbols: –, unknown; ^♂^, character states based on male specimens (female unknown or inadequately described).

Despite sharing the diagnostic characters of *Tetragoniceps* and a unique armature pattern of P2–P4, the five new species exhibit considerable interspecific variation in several appendicular characters, including the setation of the mandibular and maxillular exopods and P1enp-2, the relative length of the inner setae on the P2–P4 exopods, the morphology of P5, and the shape of the caudal ramus. These characters occur in various combinations among the Korean species, indicating considerable morphological diversity within *Tetragoniceps*.

The armature of the female P4exp-3 in the five new species consists of seven elements, including three stout (spiniform) inner elements, unlike the slender inner setae on P2–P3exp-3. In previously described *Tetragoniceps* species, the number of inner elements on female P4exp-3 has been reported as varying from one to three (Mussini et al. 2025). We re-examined the original descriptions and illustrations of previously described *Tetragoniceps* species and found that all known females possess three stout (spiniform) elements on P4exp-3. Because these stout elements arise near the distal margin of the segment, they may have been interpreted differently in previous studies. For example, *T. bergensis* was described as having an armature formula of 223 for P4exp-3 (Por 1965), in which one of the three stout elements appears to have been interpreted as an apical rather than an inner element. In the present study, these three stout (spiniform) elements are interpreted as the inner elements of the female P4exp-3. Accordingly, Table 1 provides a revised comparison of diagnostic characters in *Tetragoniceps* based on our re-examination of the original descriptions and illustrations, and interpretations adopted in this study.

The five newly described species exhibit several sexually dimorphic characters typical of *Tetragoniceps*, including elongation of the caudal ramus and the presence of an outer apophysis on the male P2 enp-2. Notably, all five new species show an additional modification of the outer distal corner of the male P4exp-2, which is either elongate and recurved (indicated by arrowhead in Fig. 8G) or inwardly concave (Figs 5D, 13G, 17E, 21D). This sexual dimorphism can also be observed in the illustrations of *T. galapagoensis* (Mielke 1989: fig. 3B), although it was not mentioned in the original description. Similar modifications of P4exp-2 have been reported in other tetragonicipitid species, including *Aigondiceps bocki* (Lang, 1948), *Diagoniceps laevis* Willey, 1930, *Neogoniceps martinezi* Fiers & de Troch, 2000, *Odaginiceps clarkae* Fiers, 1995, *O. immanis* Fiers & de Troch, 2000, *O. xamaneki* Fiers, 1995, and *Phyllopodopsyllus yucatanensis* Fiers, 1995 (Fiers 1995; Fiers and de Troch 2000). This character has received little attention from previous authors examining the *Tetragoniceps* taxonomy because the modification is subtle and difficult to observe. The occurrence of this modification in several tetragonicipitid genera suggests that it may represent a derived character state shared by a subset of genera within Tetragonicipitidae and potentially a synapomorphic trait of this lineage.

In the five newly described species, the male P4exp-3 also exhibits remarkable sexual dimorphism in the loss of the distal-most inner seta, resulting in a setal armature formula of 222. Similar sexual dimorphism is observed in other species for which males have been recorded (Nicholls 1939; Mielke 1989), although it was not always explicitly mentioned in the original descriptions. Similar loss of an inner element on the male P4exp-3 is observed in several other tetragonicipitid genera, including *A. bocki*, *D. laevis*, *D. mexicana* Fiers, 1995, *Mwania phytocola* Fiers & de Troch, 2000, *N. martinezi*, *O. immanis*, *O. xamaneki*, *P. angolensis* Kunz, 1984, *P. parastigmosus* Gómez & Morales-Serna, 2015, and *T. galapagoensis* (Mielke 1989; Fiers 1995; Fiers and de Troch 2000; Gómez and Morales-Serna 2015). However, this trait is not observed in some species whose females possess only two inner setae on P4exp-3 (e.g., *O. clarkae*; Fiers 1995). Although this character is not present in all tetragonicipitids, the loss of an inner seta on the P4exp-3 may represent another derived character state shared by several lineages within the family.

Further attention should be paid to these traits of P4exp-2 and exp-3 when analyzing the phylogenetic relationships of tetragonicipitid taxa.

Males of *Tetragoniceps* are currently known for 13 species, including the five newly described Korean species. Based on our detailed examination of these five new species, we were able to compare their morphological features and identify a common pattern in the structure of the male P5 exopod. In most species, the exopod is single-segmented and its distal margin extends into an elongate process, whereas three species (*T. bergensis*, *T. prima*, and *T. scotti*) retain the two-segmented condition without morphological deformation of the distal segment. However, the male attributed to *T. brownei* possesses a comparatively short P5 exopod (length-to-width ratio, ~ 1.0) with four elements (Wells 1967: text-fig. 60B), a condition differing substantially from that observed in other species of the genus. The male specimen additionally displays two questionable features in the antennule and caudal ramus. The elongate first antennulary segment with an outer process is regarded as a characteristic of *Tetragoniceps* in both sexes. However, the male *T. brownei* lacks the outer process and appears to have a shorter first antennulary segment than is typical of the genus (Wells 1967: text-fig. 60F). Furthermore, the position of the caudal setae is generally consistent between sexes in *Tetragoniceps*, despite variation in the shape of the caudal ramus. However, the position of the male caudal seta II attributed to *T. brownei* differs from that of the female (Wells 1967: text-figs 59D, 60E). These differences raise the possibility that the male attributed to *T. brownei* may have been misassociated with a female; therefore, the identity of this male specimen requires confirmation through re-examination.

The taxonomic position of *T. santacruzensis* is also questionable, due to the absence of the outer process on the first antennulary segment. Evaluation of its generic placement is further complicated by the lack of information on female morphology, particularly the structure of P5, because this species was described from a single male specimen. Mielke (1997) expressed reservations regarding the placement of *T. santacruzensis* within *Tetragoniceps*, although he provisionally retained the species within the genus. Gómez and Morales-Serna (2015) later discussed this uncertainty and noted similarities between *T. santacruzensis* and *Tarengoticeps yokotaanensis* Gómez & Morales-Serna, 2015, including the absence of the antennulary process. However, they considered a transfer of *T. santacruzensis* to *Tarengoticeps* Gómez & Morales-Serna, 2015 premature because the female *T. santacruzensis* and the male *T. yokotaanensis* remain unknown. The modification of the outer element on the male P3enp-2 into a recurved spine (see Mielke 1997: fig. 4A) may suggest a distinct phylogenetic affinity for *T. santacruzensis* compared to other *Tetragoniceps* species. However, a reassessment of its taxonomic position should be deferred until *T. santacruzensis* females are described.

The discovery of five previously undescribed species from Korean waters represents the first confirmed record of *Tetragoniceps* from the northwestern Pacific region and substantially extends the known distribution of the genus. This finding suggests that the diversity of *Tetragoniceps* in the northwestern Pacific has been considerably underestimated. The revised comparison of diagnostic characters presented in Table 1 and the updated identification key for *Tetragoniceps* provide a foundation for future taxonomic and phylogenetic studies of the genus.

## Supplementary Material

XML Treatment for Tetragoniceps concavus

XML Treatment for Tetragoniceps spinulifer

XML Treatment for Tetragoniceps brevisetosus

XML Treatment for Tetragoniceps unisetus

XML Treatment for Tetragoniceps protuberans

## Additional information

### Artificial Intelligence (AI) use

The authors accept full responsibility for the content of the manuscript, including the disclosure of any use of AI.

No AI tools were used in the preparation of this manuscript.

### Author ORCIDs

K. Cho https://orcid.org/0000-0002-5006-5713

J. Seo https://orcid.org/0009-0008-0912-9034

J.G. Kim https://orcid.org/0000-0001-5299-9838

J. Lee https://orcid.org/0000-0001-9004-8275

### Data availability

All of the data that support the findings of this study are available in the main text.
